# The Coevolution of RuBisCO, Photorespiration, and Carbon Concentrating Mechanisms in Higher Plants

**DOI:** 10.3389/fpls.2021.662425

**Published:** 2021-09-01

**Authors:** Peter L. Cummins

**Affiliations:** Department of Genome Sciences, John Curtin School of Medical Research, The Australian National University, Canberra, ACT, Australia

**Keywords:** ribulose-1,5-bisphosphate carboxylase/oxygenase, photorespiration, carbon concentrating mechanism, photosynthesis, evolution, homeostasis, climate change

## Abstract

Ribulose-1,5-bisphosphate (RuBP) carboxylase/oxygenase (RuBisCO) is the carbon-fixing enzyme present in most photosynthetic organisms, converting CO_2_ into organic matter. Globally, photosynthetic efficiency in terrestrial plants has become increasingly challenged in recent decades due to a rapid increase in atmospheric CO_2_ and associated changes toward warmer and dryer environments. Well adapted for these new climatic conditions, the C_4_ photosynthetic pathway utilizes carbon concentrating mechanisms to increase CO_2_ concentrations surrounding RuBisCO, suppressing photorespiration from the oxygenase catalyzed reaction with O_2_. The energy efficiency of C_3_ photosynthesis, from which the C_4_ pathway evolved, is thought to rely critically on an uninterrupted supply of chloroplast CO_2_. Part of the homeostatic mechanism that maintains this constancy of supply involves the CO_2_ produced as a byproduct of photorespiration in a negative feedback loop. Analyzing the database of RuBisCO kinetic parameters, we suggest that in genera (*Flaveria* and *Panicum*) for which both C_3_ and C_4_ examples are available, the C_4_ pathway evolved only from C_3_ ancestors possessing much lower than the average carboxylase specificity relative to that of the oxygenase reaction (*S*_C/O_=*S*_C_/*S*_O_), and hence, the higher CO_2_ levels required for development of the photorespiratory CO_2_ pump (C_2_ photosynthesis) essential in the initial stages of C_4_ evolution, while in the later stage (final optimization phase in the *Flaveria* model) increased CO_2_ turnover may have occurred, which would have been supported by the higher CO_2_ levels. Otherwise, C_4_ RuBisCO kinetic traits remain little changed from the ancestral C_3_ species. At the opposite end of the spectrum, C_3_ plants (from *Limonium*) with higher than average *S*_C/O_, which may be associated with the ability of increased CO_2_, relative to O_2_, affinity to offset reduced photorespiration and chloroplast CO_2_ levels, can tolerate high stress environments. It is suggested that, instead of inherently constrained by its kinetic mechanism, RuBisCO possesses the extensive kinetic plasticity necessary for adaptation to changes in photorespiration that occur in the homeostatic regulation of CO_2_ supply under a broad range of abiotic environmental conditions.

## Introduction

What makes Ribulose-1,5-bisphosphate (RuBP) carboxylase/oxygenase (RuBisCO) kinetic parameters the way they are? This question has now persisted for several decades, without a definitive explanation. Although RuBisCO is the principal carbon-fixing enzyme in the biosphere, its product turnover rate (circa 3s^−1^ per active site for plants) may be considered, if not particularly slow, rather unexceptional (Bar-Even et al., 2011). The primary substrate (CO_2_) must also compete for binding to the RuBP in the RuBisCO active site with the more abundant O_2_ in the atmosphere leading to photorespiration, consuming additional energy and compromising the process of photosynthesis. The combined effects of increasing population and anthropogenic climate change have motivated efforts to enhance carbon fixation in plants for increasing both agricultural crop yield and carbon sequestration generally (Niinemets et al., 2017; Andralojc et al., 2018; Erb and Zarzycki, 2018; Fernie et al., 2020; Lawson and Flexus, 2020). Although the possibility of enhancing photosynthesis by improving RuBisCO kinetic traits has been given due consideration (Whitney et al., 2011; Sharwood et al., 2016; Gomez-Fernandez et al., 2018; Wilson et al., 2018; Zhou and Whitney, 2019; Davidi et al., 2020; Lin et al., 2020; Bouvier et al., 2021), a conclusive picture of RuBisCO’s molecular mechanism (Cleland et al., 1998; Tcherkez, 2013, 2015; Cummins et al., 2018b, 2019b; Kannappan et al., 2019; Bathellier et al., 2020; Cummins and Gready, 2020) and a general consensus understanding of the observed tradeoffs between RuBisCO’s kinetic parameters remains elusive, despite having been analyzed in varying ways with the objective of gaining insights into the possible connection between evolutionary and biochemical (or catalytic) constraints (Bouvier et al., 2021). The earliest of these studies, (Tcherkez et al., 2006; Savir et al., 2010) based on general mechanistic assumptions and limited data samples, concluded that variations in the elementary rate constants must be tightly constrained by the limitations inherent in RuBisCOs kinetic mechanism, resulting in an enzyme which provides only limited scope for further optimization (Tcherkez et al., 2006; Tcherkez, 2013, 2015), while later studies of more extensive data sets have challenged this view, revealing greater flexibility (Cummins et al., 2018a, 2019a; Flamholz et al., 2019). Other studies have examined the coevolution of RuBisCO kinetics and carbon concentrating mechanism (CCMs) (Goudet et al., 2020; Iñiguez et al., 2020).

Various forms of CCMs have occurred independently and at different times in a wide range of photosynthetic organisms from diverse environments, directing the evolution of RuBisCO kinetics (Iñiguez et al., 2020). In higher plants, the vast majority follow the C_3_ photosynthetic pathway initiated by CO_2_ fixing to the bound form of activated RuBP substrate (Cleland et al., 1998) which proceeds through hydrolysis to break down into two molecules of the 3-carbon compound 3-phosphoglyceric acid (3PGA). The C_4_ pathway, although present in only about 8,000 species (Sage, 2016), including some important agricultural crops (maize and sorghum), accounts for about 25% of terrestrial photosynthesis. In C_4_ photosynthesis, CO_2_ is initially converted by carbonic anhydrase (CA) to bicarbonate which is fixed by phosphoenolpyruvate carboxylase into oxaloacetate. Oxaloacetate is converted into malate (4-carbon compound) or aspartate for diffusion into the bundle sheath cells where they are decarboxylated in high concentrations and the released CO_2_ is then, as in C_3_ photosynthesis, fixed into 3PGA by RuBisCO. By increasing CO_2_ supplies and thereby suppressing photorespiration, plant CCMs began evolving from C_3_ species, probably in the early Oligocene (circa 30ma) in response to decreasing CO_2_ levels, as an efficient way of increasing photosynthesis in the more challenging environmental conditions (Sage, 2004). The evolutionary success of C_4_ photosynthesis and the observation of increased carbon assimilation in some C_3_ crops under elevated CO_2_ (Xu et al., 2016; Thompson et al., 2017) have stimulated research into the possibility of incorporating CCMs into C_3_ plant species via genetic modification (McGrath and Long, 2014; Jurić et al., 2019; Kubis and Bar-Even, 2019; Atkinson et al., 2020).

Selective abiotic forces have determined the evolution of C_4_ photosynthesis over a number of distinct phases (Sage et al., 2018). C_2_ photosynthesis, a crucial step in the early stages of this evolution (Sage et al., 2012; Bräutigam and Gowik, 2016), is characterized by the formation of a photorespiratory CO_2_ pump that utilizes the two-carbon compound, glycine, to transport and concentrate the photorespiratory CO_2_ into the bundle sheath cells where it can be re-fixed by RuBisCO. The processes involved in elevating levels of photorespiration relative to photosynthesis in C_3_ plants are, therefore, a fundamentally important selection factor initiating the evolution to C_4_ photosynthesis (Sage et al., 2018). This ratio can be modelled using the rate, or velocity, *v*, of the oxygenase relative to the carboxylase catalyzed reaction, given by (Laing et al., 1974; Jordan and Ogren, 1984)

(1) $$v_{O/C}=\frac{v_{O}}{v_{C}}=\frac{O}{S_{C/O}C}$$

where *O* and *C* are, respectively, the O_2_ and CO_2_ concentrations at carboxylation sites, and *S*_C/O_ is the specificity of the carboxylase relative to the oxygenase reaction which can be expressed in various algebraic forms as

(2) $$S_{C/O}=\frac{S_{C}}{S_{O}}=\frac{V_{C/O}}{K_{C/O}}=\frac{V_{C}}{V_{O}}\frac{K_{O}}{K_{C}}=V_{C/O}K_{O/C}$$

In an obvious notation, the RuBisCO kinetic parameters *V* and *K* denote the maximum catalytic (turnover) rates (*V*_max_ or *k*_cat_) and Michaelis constant (*K*_M_), respectively. A recent data compilation contains RuBisCO kinetic parameters (at 25° C) from over 300 species (Flamholz et al., 2019), with more than 50% derived from higher plants. Of these plant species, the ubiquitous C_3_ plants constitute by far the largest sample, followed by around 40 examples of C_4_ plants, while samples of C_3_-C_4_ intermediate (extant plants exhibiting C_2_ photosynthesis on the pathway to C_4_) and some C_4_-like plants from *Flaveria*, a genus adopted as a model for the evolutionary pathway of C_4_ photosynthesis (McKown et al., 2005; Kapralov et al., 2011; Sage et al., 2013; Schulze et al., 2013; Mallmann et al., 2014), and other miscellaneous plants, including examples of CCM-containing plants that follow the crassulacean acid metabolism pathway, are fewer in number.

The reduction of atmospheric CO_2_ levels and associated increase in O_2_ would have undoubtedly enforced adaptation of plants to increased photorespiration over geological time. Increasing temperature also increases *v*_O/C_ (Jordan and Ogren, 1984) by decreasing both *S*_C/O_ and the ratio of substrate concentrations, *C*/*O*, and is an important environmental factor driving the evolution of the C_4_ pathway (Sage et al., 2018). However, while photorespiration is not particularly advantageous to C_3_ plants due to net loss of carbon, it is also important to recognize that a significant amount of photorespiratory CO_2_ can feed into chloroplasts (Sage and Sage, 2009; Tholen and Zhu, 2011; Busch et al., 2013), increasing the potential for the recapture of carbon for photosynthesis and thus facilitating evolution along the C_4_ pathway. This creates a negative feedback loop that mitigates the photorespiratory response and thus limiting the increases in *v*_O/C_. Moreover, the combined action of CA (releasing CO_2_ from bicarbonate) and photorespiration have been postulated to form the basis of a homeostatic mechanism that ensures a stable supply of CO_2_ to RuBisCO, essential for the energy efficient maintenance of photosynthesis (Riazunnisa et al., 2006; Igamberdiev and Roussel, 2012; Igamberdiev, 2015).

While the importance of abiotic environmental conditions leading to carbon restriction (reduced atmospheric CO_2_, higher temperatures, and lack of water) in driving evolution of the photorespiratory CO_2_ pump is well understood (Sage et al., 2018), the role of RuBisCO kinetic variability, which underpins *S*_C/O_, warrants further critical investigation (Sage, 2013; Sage et al., 2018). In the present study, we have attempted to delineate possible coevolutionary relationships between RuBisCO, photorespiration, and CCMs by analyzing RuBisCO kinetic parameter data for higher plant species derived from the most recent compilation (Flamholz et al., 2019). In two genera, *Flaveria* and *Panicum*, the results suggest that in addition to abiotic conditions that increase photorespiration by lowering *S*_C/O_, much lower than average *S*_C/O_ in the C_3_ populations could also be a critically important precondition in C_4_ evolution. In contrast, C_3_ plants (e.g., *Limonium*) that have adapted to extreme abiotic environments are typically characterized by higher than average *S*_C/O_ (Galmés et al., 2005), which compensates for the lower levels of photorespiratory CO_2_ (Equation 1) through higher CO_2_ affinity.

## Statistical Methods

The data sets for our analysis were accessed from the compilation by Flamholz et al. (2019). Where there are multiple entries per species in this database, these were averaged prior to statistical analysis. The complete list of species and associated kinetic parameters used in the analysis is provided in the Supplementary Material (Supplementary Tables S1 and S2). It is worth noting here that correlations between these kinetic parameters have been recently analyzed using Phylogenetic Generalized Least Squares (PGLS) as opposed to standard least-squares regression (Bouvier et al., 2021). Standard regression analysis assumes independence of the residuals, which may not necessarily be true when looking for correlations between traits in evolutionary biology as those taxa with a more common ancestor (more related) would exhibit similar traits and hence dependent residuals. PGLS methods are often used to account for such dependencies (for a general overview of PGLS methodology, see Mundry, 2014). The PGLS study by Bouvier et al., (2021) suggests that correlations between RuBisCO kinetic parameters are over estimated by standard regression; i.e., a significant phylogenetic signal is present. Nevertheless, the covariance between *K*_C_ and *V*_C_ appears not affected, which is perhaps not surprising given the interdependence between *K* and *V* (Briggs and Haldane, 1925). In view of this underlying covariance present in the enzyme kinetics, which is quite distinct from phylogenetic and catalytic constraints, standard linear least-squares regression analysis was carried out on the total C_3_ (excluding *Limonium*), C_3_ *Limonium* collected from high stress (high temperature and water restricted) habitat (Galmés et al., 2014), *Flaveria* sample and total C_4_ samples. The vast majority of grass species are split fairly evenly between the closely related Panicoideae, Arundinoideae, Chloridoideae, Micrairoideae, Aristidoideae, and Danthonioideae (PACMAD) and Bamboos, Oryzoideae, and Pooideae (BOP) sister clades. Among grasses, C_4_ photosynthesis has evolved from C_3_ only in PACMAD species (for an overview, see Christin et al., 2013). As both clades are relatively well represented in the kinetic data, correlations are also examined separately for *Oryza*, *Aegilops*, *Puccinellia* (all BOP), and *Panicum* (PACMAD) species.

The estimation of effect sizes (Cumming, 2012) of primary interest in our analysis is both the differences between mean kinetic parameters and, in particular, the differences between coefficients obtained from linear least-squares regression. As by definition *K*_M_ is expressed as an explicit function of *V*_max_ (Briggs and Haldane, 1925), they are not independent variables. The Michaelis constant for the carboxylase and oxygenase reactions can be written in the general linear in *V*_max_ form (Cummins et al., 2018a),

(3) $$K_{M}=mV_{\max}+b$$

The coefficient of the *K*_M_ intercept and coefficient of *V*_max_ obtained by linear regression of the data can be interpreted as the sample mean values of *b* and *m*, respectively, which are functions of the rate constants for the elementary steps in the kinetic mechanism (Cummins et al., 2018a). Prior linear regression analysis of the explicit dependence of *K*_M_ on *V*_max_ indicates that *K*_M_ is to some extent dependent on the value of *b*, which is a function of the dissociation rates (among other rate constants) of the CO_2_ and O_2_ gas substrates (Cummins et al., 2018a, 2019a).

The margin of error in the difference between two means is estimated using confidence intervals (CIs), calculated from the standard errors of the means (SE). For two independent samples of size *n*_1_ and *n*_2_ with means M_1_ and M_2_ (e.g., regression coefficients for C_3_ and C_4_ samples), the combined SE for the difference in the means (∆M) can be obtained by quadrature, giving the CI as follows:

(4) $$\mathrm{CI}=z\sqrt{{\left({\mathrm{SE}}_{1}\right)}^{2}+{\left({\mathrm{SE}}_{2}\right)}^{2}}$$

Alternatively, quadrature may be used with fractional errors to express differences as a percentage, as

(5) $$\mathrm{CI}=z\frac{M_{2}}{M_{1}}\sqrt{{\left(\frac{{\mathrm{SE}}_{1}}{M_{1}}\right)}^{2}+{\left(\frac{{\mathrm{SE}}_{2}}{M_{2}}\right)}^{2}}$$

In Equations (4) and (5), Student’s inverse cumulative distribution function (*t*^−1^) is given by

(6) $$z=t^{-1}\left(\alpha ,n_{1}+n_{2}-2\right)$$

The CIs can be readily interpreted in terms of *p* values. If │∆M│> CI, the difference may be considered “statistically significant” (null hypothesis may be rejected) at *α*=*p*. We consider that only very small *p* values (at best, *p* <0.01) should provide a reliable foundation for rejecting a null hypothesis (Cumming, 2012). The statistical analysis was generated using the Real Statistics in Excel software package (Zaiontz, 2020).

## Results

Mean values of the RuBisCO kinetic parameters are given in Table 1 for the total samples of C_3_ (excluding *Limonium*), C_3_ *Limonium* and C_4_ plants. Since *K*_C_ differences increase in proportion to the *V*_C_ (probably by around 40% of mean C_3_ values), carboxylase specificity (*S*_C_=*V*_C_/*K*_C_) does not make a significant contribution to differences in mean *S*_C/O_. The difference in *S*_C/O_ between C_3_ and C_4_ is therefore determined largely by *S*_O_, primarily through the increase maximum turnover rate for the oxygenase reaction, *V*_O_. The transition from C_3_ to C_4_ plants is accompanied by an estimated 10–25% decrease in mean *S*_C/O_, due overwhelmingly to changes in the kinetics of the oxygenase reaction alone. Overall, the results in Table 1 are similar to those in the PGLS study (Bouvier et al., 2021); in C_4_ species, *S*_C/O_ is lower than in C_3_, and both *V*_C_ and *K*_C_ are higher than in C_3_ species. The sample of C_3_ *Limonium* species differs from the main C_3_ sample in both higher *S*_C_ and *S*_O_ means, determined largely by decreases in *K*_C_ and *K*_O_. The resulting mean *S*_C/O_ in *Limonium* is 15% higher than the mean of the C_3_ sample.

**Table 1:** Sample sizes (*n*) and mean values of RuBisCO kinetic parameters: maximum turnover rates, *V*_max_ (s^−1^), Michaelis constants, *K*_M_ (μM), and specificities, *S*=*V*_max_/*K*_M_ (s^−1^.mM^−1^) and relative specificities *S*_C/O_ with standard errors in the means (SE) for C_3_, C_3_ *Limonium*, and C_4_ plants.

C_3_^a^				C_3_ *Limonium*			C_4_			C_4_−C_3_^b^	
*n*		Mean	SE	*n*	Mean	SE	*n*	Mean	SE	Mean	%C_3_
*V*_C_	126	3.22	0.08	17	2.76	0.13	26	4.42	0.23	1.27^***^	40.3 ± 16.0
*K*_C_	124	16.0	0.5	17	8.61	0.23	30	21.0	1.4	5.9^***^	39.4 ± 19.9
*S*_C_	116	213	6	17	320	9	25	241	19	14	6.1 ± 17.7
*V*_O_	94	1.01	0.03	14	1.09	0.05	21	1.46	0.13	0.43^**^	42.2 ± 25.5
*K*_O_	108	497	14	17	380	12	25	512	54	31	6.5 ± 22.6
*S*_O_	106	2.03	0.05	14	2.92	0.13	25	3.02	0.26	0.88^***^	41.2 ± 24.6
*S*_C/O_	126	97.8	0.8	14	112	1	31	81.0	1.5	−18.1^***^	−18.3 ± 6.2

Differences in the means (C_4_−C_3_) are also expressed as percentage of C_3_ values (%C_3_) with 95% confidence (*α*=0.05) intervals.

** *p* <0.01;

*** *p* <0.001.

a Excluding C_3_ *Limonium*.

b Including C_3_ *Limonium*.

**Figure 1:**
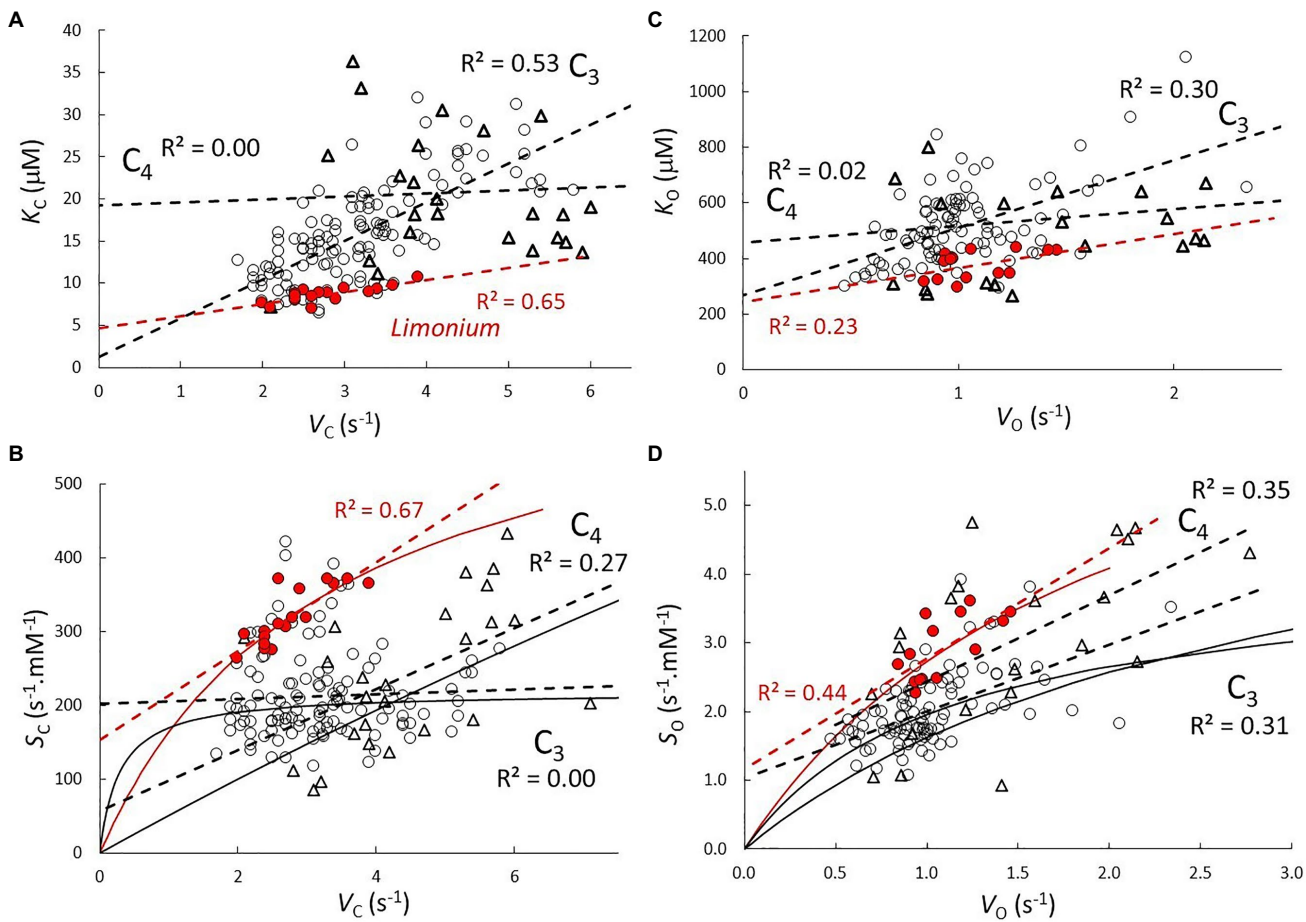
Correlations between parameters *V*_max_, *K*_M_, and *S* from **(A,B)** carboxylase and **(C,D)** oxygenase reactions. Scatter plots and lines (dash) of best fit with *R*^2^ for C_3_ (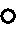), C_3_ *Limonium* (highlighted in red), and C_4_ (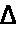) plant RuBisCO parameters. Solid lines in (**B**; *S*_C_ vs. *V*_C_) and (**D**; *S*_O_ vs. *V*_O_) are predictions of the actual curves (Equation 7) based on coefficients (Table 2) obtained from regression of data in **(A,C)**, respectively.

**Figure 2:**
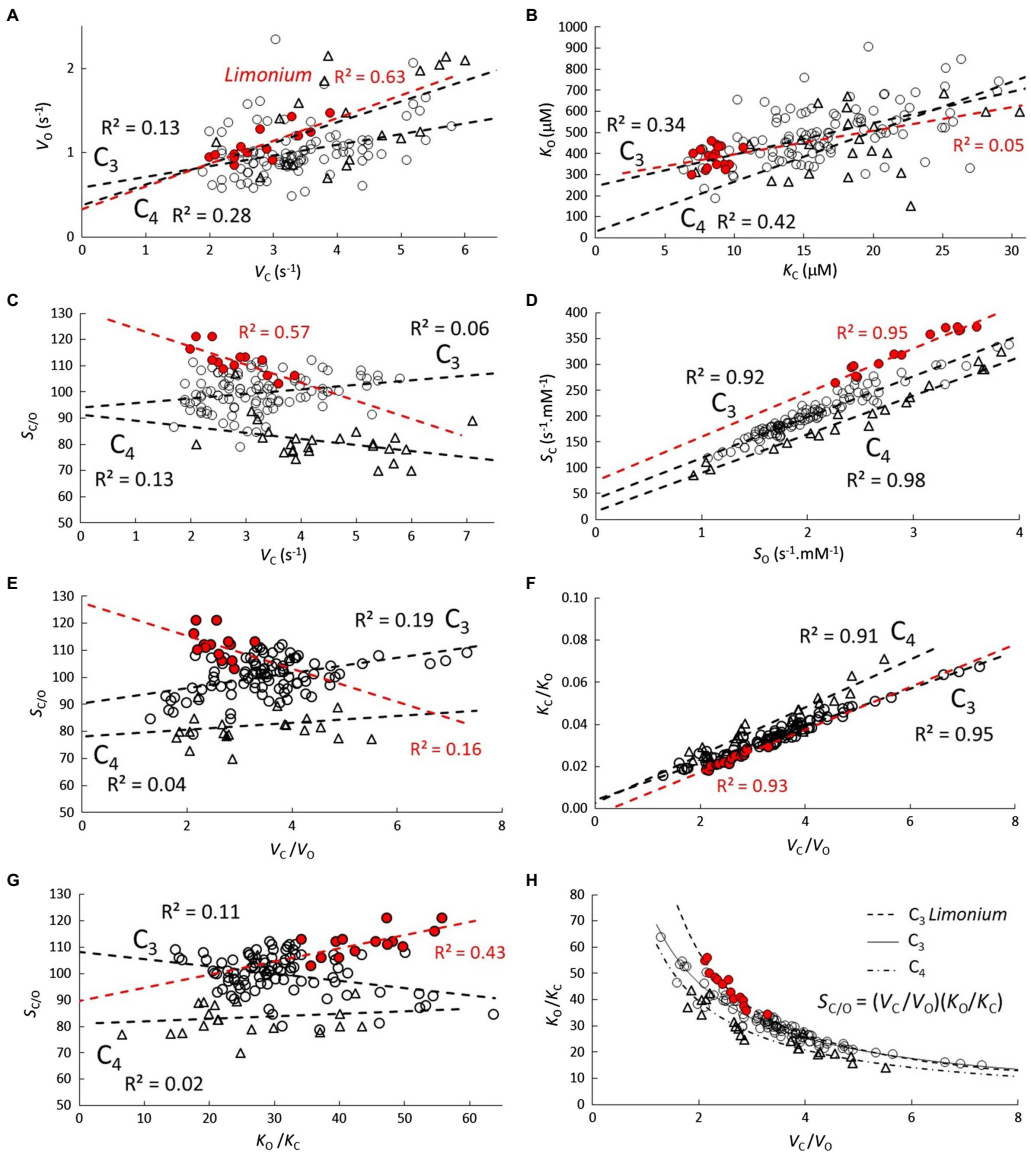
Correlations **(A-H)** between carboxylase and oxygenase kinetic parameters. Scatter plots and lines of best fit with *R*^2^ for C_3_ (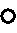), C_3_ genus *Limonium* (highlighted in red), and C_4_ (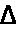) plant RuBisCO parameters.

Regression coefficients and their standard errors are given in Table 2 for the C_3_ (excluding *Limonium*), C_3_ *Limonium*, and C_4_ samples. Scatter plots of the data and lines of best fit with *R*^2^ are shown in Figure 1 for correlations between parameters from the same reaction (carboxylase or oxygenase), and in Figure 2 for correlations between carboxylase and oxygenase parameters. Results obtained for carboxylase (Figure 1A) indicate differences between carboxylation parameters in C_3_, C_3_ *Limonium*, and C_4_ plant RuBisCOs. On average, *b* is negligible (when compared to the product, *mV*_max_) in the C_3_ plant sample. For the carboxylase reaction, the gradients (*m*) and intercept (*b*) of the lines of best fit decrease and increase, respectively, from C_3_ to C_3_ *Limonium*, to C_4_. Also of note are the lower *R*^2^ values (higher variance) for the C_3_ and C_4_ samples as compared to C_3_ *Limonium* sample.

**Table 2:** Results of linear regression analysis for C_3_ (excluding *Limonium*), C_3_ *Limonium*, and C_4_ plants.

		C_3_		C_3_ *Limonium*		C_4_		C_4_-C_3_	C_4_-*Lim.*	C_3_-*Lim.*
Figure		coeff.	SE	coeff.	SE	coeff.	SE	coeff.	coeff.	coeff.
1A	*K*_C_ (0)	1.31	1.37	4.67^***^	0.76	19.2^**^	6.2	17.9^**^	14.6^*^	−3.4^*^
	*V*_C_	4.58^***^	0.41	1.42^***^	0.27	0.36	1.35	−4.22^*^	−1.06	3.2^***^
1B	*K*_O_ (0)	269^***^	42	243^**^	71	457^*^	171	188	214	26.3
	*V*_O_	242^***^	41	123	64	60	109	−182	−63	119
1C	*S*_C_ (0)	203^***^	21	154^***^	31	57	66	−146^*^	−97	49
	*V*_C_	3.1	6.3	60.1^***^	10.9	41.4^**^	14.3	38.2^*^	−18.8	−57.0^***^
1D	*S*_O_ (0)	1.03^***^	0.16	1.17	0.58	1.19	0.62	0.16	0.02	−0.14
	*V*_O_	0.97^***^	0.15	1.61^**^	0.52	1.25^**^	0.65	0.17	−0.35	−0.63
2A	*V*_O_ (0)	0.58^***^	0.12	0.33	0.17	0.37	0.41	−0.21	0.04	0.25
	*V*_C_	0.13^***^	0.03	0.27^***^	0.06	0.25^*^	0.09	0.12	−0.02	−0.14
2B	*K*_O_ (0)	248^***^	36	283^*^	116	30	125	−218	−253	−35
	*K*_C_	14.9^***^	2.0	11.3	13.4	23.7^***^	5.8	8.8	12.3	3.6
2C	*S*_C/O_ (0)	94.1^***^	2.3	131^***^	5	91.4^***^	5.5	−2.8	−39.7^***^	−36.9^***^
	*V*_C_	1.72^*^	0.70	−6.87^**^	1.71	−2.31	1.21	−4.02^**^	4.56^*^	8.58^***^
2D	*S*_C_ (0)	39.6^***^	4.7	74.4^***^	16.5	15.3	8.0	−24.3^**^	−59.2^**^	−34.9^*^
	*S*_O_	79.2^***^	2.2	85.6^***^	5.6	74.7^***^	2.4	−4.4	−10.9	−6.5
2E	*S*_C/O_ (0)	90^***^	2	127^***^	11	78^***^	5	12^*^	−49^***^	−37^***^
	*V*_C_/*V*_O_	2.82^***^	0.61	−6.11	4.10	1.27	1.52	−1.55	7.38	8.93^*^
2F	*K*_C_/*K*_O_ (0)^a^	3.99^***^	0.79	2.82	2.77	−3.03	2.11	−1.17	11.7^**^	5.85^*^
	*V*_C_/*V*_O_^a^	8.8^***^	0.2	11.3^***^	0.8	10.1^***^	0.8	2.50^**^	2.83^*^	1.17
2G	*S*_C/O_ (0)	108^***^	3	89.6^***^	7.5	81.6^***^	4.5	−26.7^***^	−8.1	18.6^*^
	*K*_O_/*K*_C_	−0.27^***^	0.08	0.50	0.17	0.04	0.16	0.32	−0.46	−0.77^***^

The coefficients of the independent variable (e.g., *V_C_* in Figure 1A) and intercept on the dependent variable axis (0) correspond to the sample mean values of *m* and *b*, respectively, in Equation 3, and are dependent on details of the enzyme’s kinetic mechanism.

* *p* <0.05;

** *p* <0.01;

*** *p* <0.001.

a ×10^3^.

The actual trend lines for *S* vs. *V*_max_ (Figures 1B,D) can be calculated by expressing the specificity (*V*_max_/*K*_M_) in the general hyperbolic in *V*_max_ form

(7) $$S=\frac{V_{\max}}{mV_{\max}+b}$$

and substituting *m* and *b* with the corresponding *K*_M_ vs. *V*_max_ regression coefficients given in Table 2. *S* increases from zero with increasing *V*_max_, reaching the asymptotic limit value *m*^−1^ when *mV*_max_>>*b*. It is also clearly apparent that the linear equation obtained from regression of the *Limonium* data (with higher *R*^2^) is tangential to the predicted curves in the vicinity of the data points. That the coefficient corresponding to *m*^−1^ should approximate the mean value of *S* is easily verified by comparing the results in Tables 1 and 2, justifying the underlying assumptions of the regression analysis. The rate at which this limit is reached depends on *m*.*b*^−1^ (verifiable from the corresponding coefficients in Table 2). For carboxylase, we find that the rate of increase in *S* with respect to increasing *V*_max_ is relatively fast in the sample of C_3_ plants, while significantly slower for C_3_ *Limonium*, and in C_4_ plants, the limiting value is reached very slowly. Unlike carboxylase, the regression coefficients obtained for the oxygenase reaction (Figure 1C) indicate little difference between the C_3_, C_3_ *Limonium*, and C_4_ plant groupings. In contrast to the C_3_ sample which shows practically no correlation between *S*_C_ and *V*_C_ (Figure 1B), all groups exhibit positive correlations for *S*_O_ vs. *V*_O_ (Figure 1D).

Varying degrees of correlation are evident between carboxylase and oxygenase kinetic parameters (Figure 2). Positive correlations are observed in both *V*_O_ vs. *V*_C_ (Figure 2A) and *K*_O_ vs. *K*_C_ (Figure 2B), although the differences between C_3_, C_3_ *Limonium* and C_4_ are not significant. Nevertheless, differences between the three groups do become apparent in many of the other correlations. In particular, very strong (*R*^2^>0.9) *S*_C_ vs. *S*_O_ correlations (Figure 2D) clearly distinguish the three groups. The different forms in Equation (2) suggest similarly strong linear correlations should also be obtained for *V*_C_/*V*_O_ vs. *K*_C_/*K*_O_ (Figure 2F). Inverting the linear equations obtained from the *V*_C_/*V*_O_ vs. *K*_C_/*K*_O_ regression provide hyperbolic-like functions which accurately predict the reciprocal *V*_C_/*V*_O_ vs. *K*_O_/*K*_C_ plots (Figure 2H) for each of the three group samples. Other correlations, *S*_C/O_ vs. *V*_C_, *S*_C/O_ vs. *V*_C/O_, and *S*_C/O_ vs. *K*_O/C_, relevant for the discussion and interpretation of Equation (2) are also shown in Figure 2.

**Figure 3:**
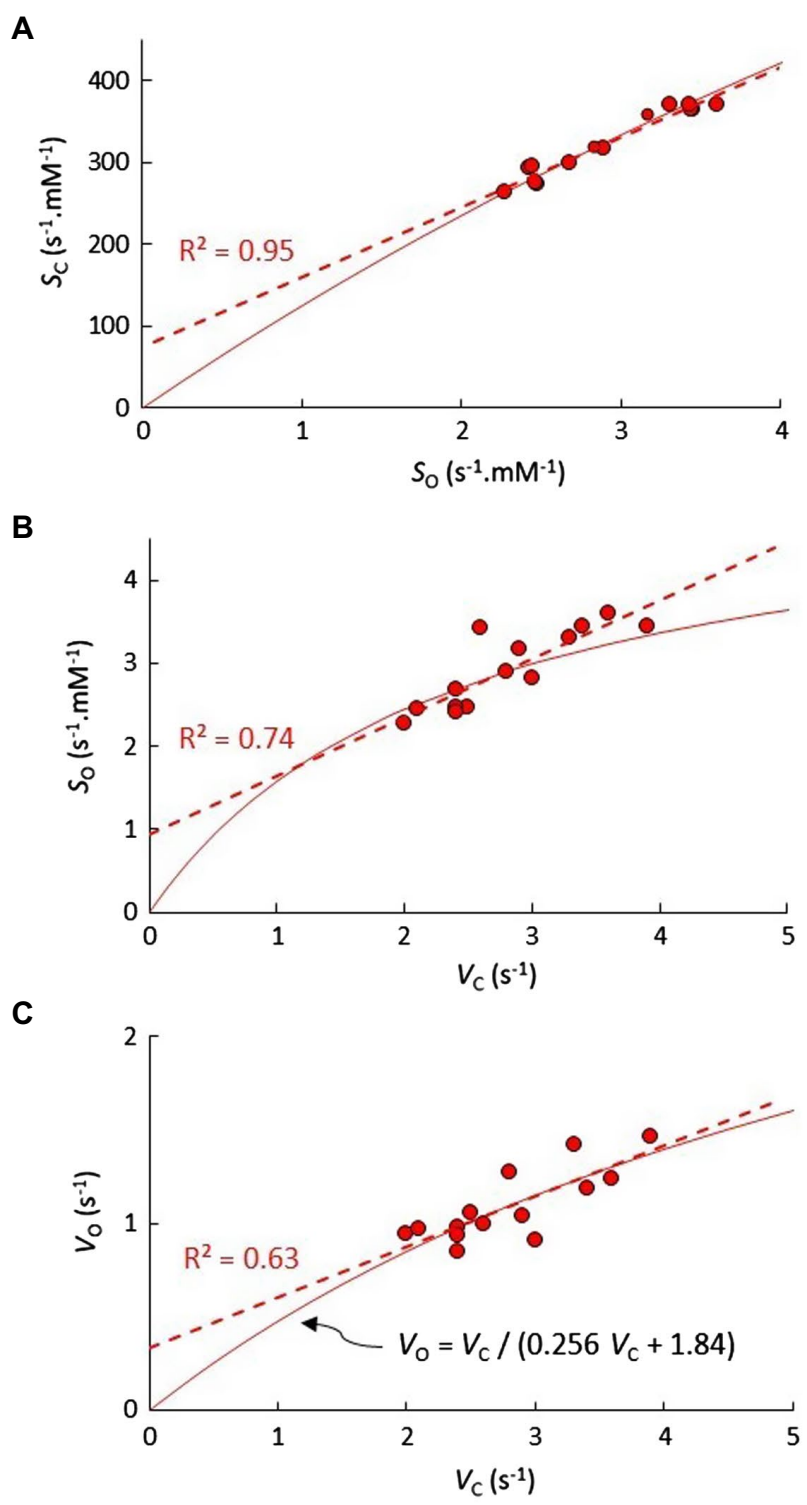
The predicted trend (solid line) in **(A)** *S*_C_ vs. *S*_O_ for genus C_3_ *Limonium*, determined using the set of parametric equations describing the trends in *S*_C_ vs. *V*_C_ (Figure 1B) and **(B)** *S*_O_ vs. *V*_C_. In **(B)**, the solid trend line for *S*_O_ vs. *V*_C_ is determined by substituting **(C)** the hyperbolic equation obtained from the nonlinear least squares fit of the *V*_O_ vs. *V*_C,_ data for *V*_O_ in the equation for the predicted *S*_O_ vs. *V*_O_ curve (Figure 1D).

As shown in Figure 1, the correlation coefficients obtained from the linear regression of *K*_M_ vs. *V*_max_ in Table 2 can be used to predict the correlations in *S* vs. *V*_max_. The relatively high level of correlation in the *Limonium* data suggests that an accurate estimation of the curve for *S*_C_ vs. *S*_O_ should be obtainable using parametric equations derived from *S*_C_ vs. *V*_C_ and *S*_O_ vs. *V*_C_. Equations for *S*_C_ vs. *V*_C_ and *S*_O_ vs. *V*_O_ are already defined (Figures 1B,D), and an equation (Figure 3C) for *V*_O_ in terms of *V*_C_ derived from nonlinear regression of *V*_O_ vs. *V*_C_ (Figure 2A) can be substituted for *V*_O_ in the equation for *S*_O_ vs. *V*_O_, yielding the desired equation for *S*_O_ vs. *V*_C_ (Figure 3B). In each case, the linear equations obtained from regression of data in Figure 3 are quite clearly tangential to the predicted curves in the vicinity of the data points. Moreover, the predicted curve for *S*_C_ vs. *S*_O_ (Figure 3A) is in fact very nearly linear.

**Figure 4:**
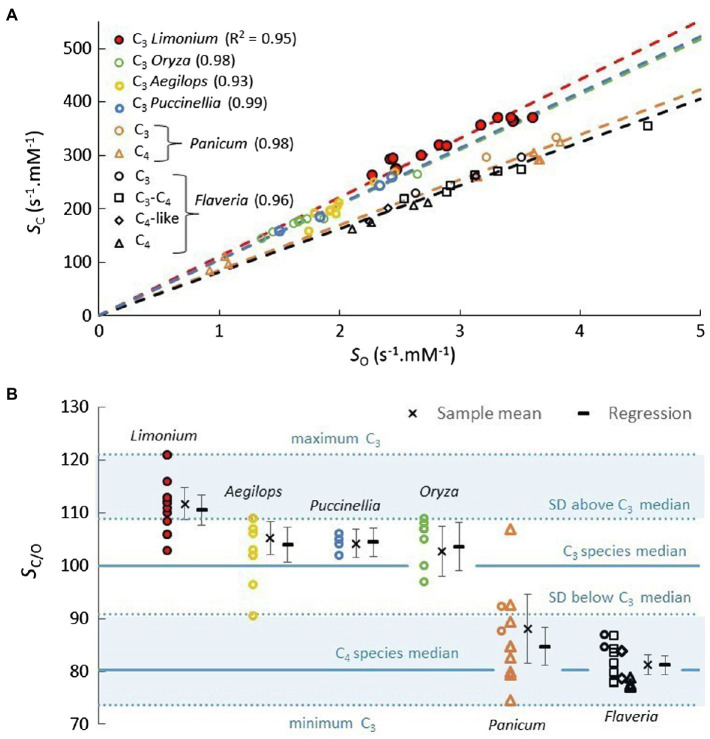
**(A)** Plots of *S*_C_ vs. *S*_O_ for genera with lines of best fit passing through *S*_C_=0. **(B)** Diagram illustrating the variance of *S*_C/O_. The sample means are well approximated by the gradients (regression coefficient) of the trend lines in **(A)**. The error bars correspond to 95% (*α*=0.05) confidence intervals. The lower shaded area, less than one SD below the median, represents around 10% of the total C_3_ species sample, with a mean *S*_C/O_ of 83.4 (*V*_C_=2.85s^−1^, *K*_C_=12.1μM, *S*_C_=245s^−1^.mM^−1^, *V*_O_=1.43s^−1^, *K*_O_=489μM, *S*_O_=2.85s^−1^.mM^−1^).

For each genus, the near-linear trend predicted in Figure 3A suggests performing the linear regression for *S*_C_ vs. *S*_O_ with the intercept fixed at zero (Figure 4A). Further, the gradient (regression coefficient) of the regression line in this way quite accurately predicts the sample *S*_C/O_ means for each of the genera (Figure 4B), which again validates our basic assertion that the coefficients of the linear regression should correspond to sample means (Cummins et al., 2018a, 2019a). Examples of genera with data for more than a few species are rather limited. In addition to *Limonium*, for which there is a reasonably sized sample (Table 1), C_3_ plant data are available for numbers of BOP species from *Oryza*, *Aegilops*, and *Puccinellia*. Both C_3_ and C_4_ plant data are available for *Panicum*, while *Flaveria* is the only genus for which there are parameters for C_3_, C_4_, and transitional (C_3_-C_4_ and C_4_-like) species (Supplementary Tables S1 and S2). The results in Figure 4A reveal a high level (*R*^2^>0.90) of *S*_C_ vs. *S*_O_ correlation between species within the various genera, irrespective of photosynthetic (C_3_ or C_4_) pathway. The distribution of *S*_C/O_ in these genera is illustrated more clearly in Figure 4B. For the vast majority of *Panicum* and *Flaveria* species (both C_3_ and C_4_), the *S*_C/O_ is more than one SD below the median of the total C_3_ sample.

**Figure 5:**
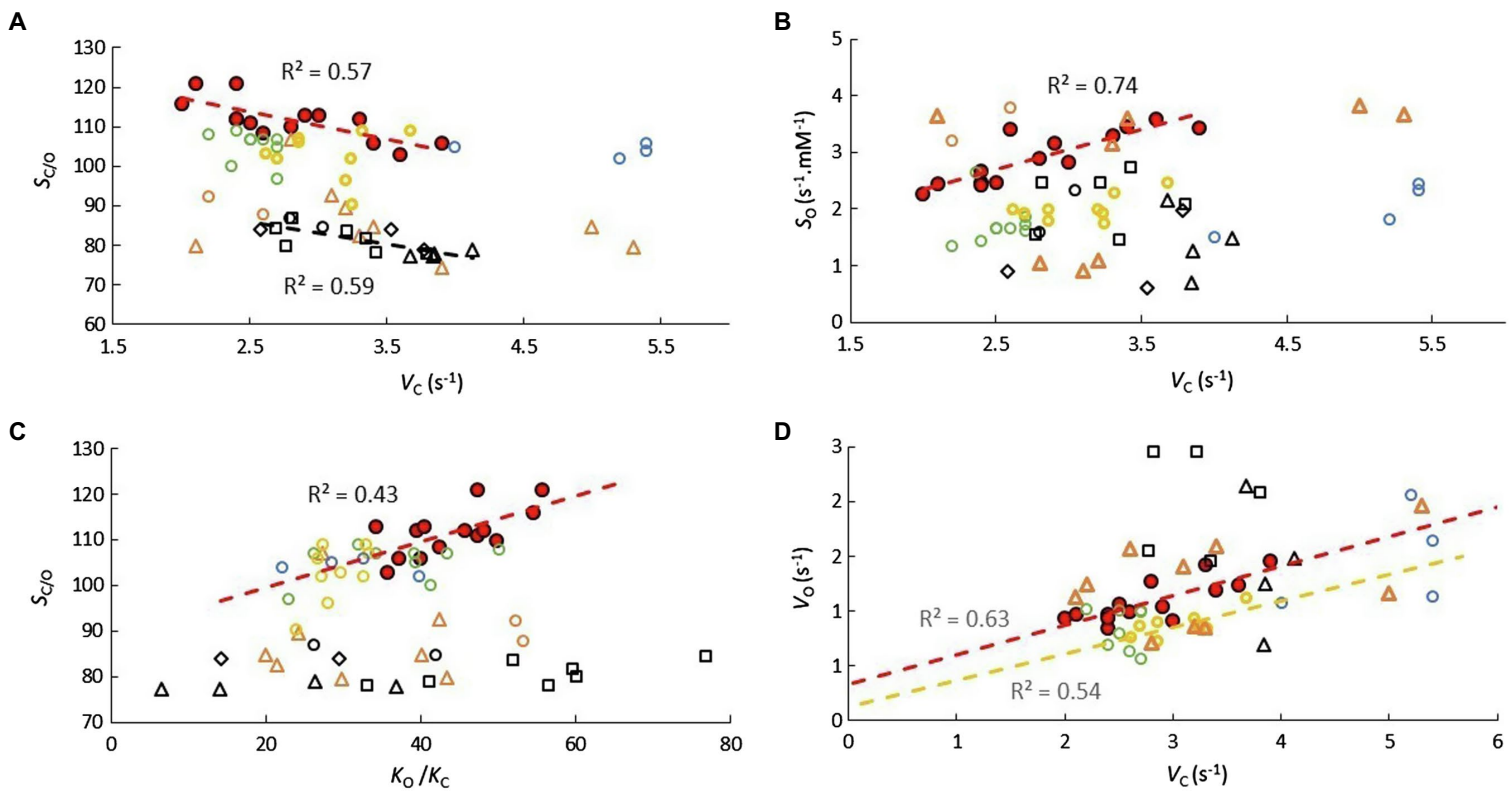
Scatter plots of **(A)** *S*_C/O_ vs. *V*_C_, **(B)** *S*_O_ vs. *V*_C_, **(C)** *S*_C/O_ vs. *K*_O_/*K*_C_, and **(D)** *V*_O_ vs. *V*_C_ for various genera. Description of symbols used is the same as in Figure 4.

There are some other correlations of note that distinguish the various genera (Figure 5). Significant *S*_C/O_ vs. *V*_C_ correlations are obtained for both *Limonium* and *Flaveria* (5A), but only for *Limonium* in *S*_O_ vs. *V*_C_ (5B). In Figure 5C, increased *S*_C/O_ in *Limonium*, *Oryza*, *Aegilops*, and *Puccinellia* (exclusively C_3_) species correlates with increased CO_2_ affinity (*K*_O/C_), while for *Panicum* and *Flaveria* (predominantly C_4_ species), increased *K*_O/C_ has little tendency to increase *S*_C/O_, which appears to be true generally for species that have evolved C_4_ photosynthesis (Figure 2G).

## Discussion

### Photorespiration in the Evolution of RuBisCO and C_4_ Photosynthesis

The evolutionary pathway to C_4_ photosynthesis necessitates extensive structural, biochemical, and genetic modifications in the ancestral C_3_ plants (Gowik et al., 2011). Considering the current understanding of C_4_ evolution has been achieved through a broad multidisciplinary approach (Sage et al., 2018), it is noteworthy that the numbers of published RuBisCO kinetic studies over the last decade have shown a steep decline (Hanson, 2016). C_4_ photosynthesis is a prime example of convergent evolution (Blount et al., 2018), having arisen on at least 66 occasions over the past 30ma (Sage et al., 2011), producing many thousands of species spread globally over many diverse plant families (Sage, 2016). Despite being the most extensively studied enzyme, at least in terms of kinetics (Jeske et al., 2019), compilations of the C_4_ RuBisCO kinetic parameters (Flamholz et al., 2019) barely scratch the surface of the total global C_4_, and for that matter, C_3_ populations.

Despite the large gaps in the available data, what exactly can be understood in relation to the coevolution of RuBisCO kinetics and C_4_ photosynthesis? Table 1 shows clear differences between sample means of most C_3_ and C_4_ kinetic parameters. However, it seems that the fundamental question is whether, or to what extent, these differences arise from adaptation over time along the evolutionary C_4_ pathway, or they are mostly traits inherited, with minimal change, from the ancestral C_3_ species? Comparing sample means can only provide the answer if the evolving C_4_ plants were randomly selected from the broader C_3_ population. The evidence suggests this may not be the case, as both *Flaveria* and *Panicun* C_3_ species exhibit *S*_C/O_ values much lower than the C_3_ average (Figure 4B), which could well be an advantage in the early-stage evolution of C_4_ plants, as the first stages of C_4_ evolution involve establishment of the photorespiratory CO_2_ pump (C_2_ photosynthesis). Given this initial requirement for photorespiratory CO_2_, it would not be unexpected to find positive selection of C_3_ species with low *S*_C/O_.

While C_4_ evolution may have followed a number of different pathways (Schüssler et al., 2017), in the *Flaveria* model, C_2_ photosynthesis is associated with intermediate C_3_-C_4_ species, transitioning to C_4_-like in the final “optimization” stages (Sage et al., 2018); phylogenetic analysis of C_3_, transitional and C_4_ species in *Flaveria* (Kubien et al., 2008; Kapralov et al., 2011) reveals correlations with variation in kinetic parameters. While not so apparent in the combined C_3_ and C_4_ samples (Figure 2C), the expected negative *S*_C/O_ vs. *V*_C_ trend (Tcherkez et al., 2006) found in *Flaveria* (Kubien et al., 2008) is reproduced in Figure 5A. One interpretation of this result is that increased CO_2_ and decreased O_2_ levels favor selection of RuBisCO with lower *S*_C_ and higher *V*_C_, with little change in oxygenase kinetics. This appears to be supported in Figure 5B which reveals that the trend is determined exclusively by decreasing *S*_C_ (increasing *K*_C_), as *S*_O_ vs. *V*_C_ in *Flavaria* shows no correlation, so that by inference the observed reduction of *S*_C/O_ with increasing *V*_C_ in *Flaveria* must arise from *S*_C_ alone. However, as we explain below, these trends in the adaptation of RuBisCO would most likely have arisen much later in the evolution of C_4_, well after the establishment of a functional CCM.

There is no difference between the C_3_ and C_4_ mean *S*_C_ to suggest there is adaptation toward decreased carboxylation (*K*_C_) in favor of speed (*V*_C_); increased *V*_C_ alone causes the increase in *K*_C_, maintaining the stability in *S*_C_ (Table 1). Rather, the difference between C_3_ and C_4_ mean *S*_C/O_ stems from *S*_O_ alone, predominately through higher oxygenase turnover (*V*_O_). If sampling is restricted to the C_3_ species with *S*_C/O_ less than one SD below the median (Figure 4B), the resulting mean values of *V*_O_ (1.43s^−1^) and *K*_O_ (489μM) are comparable to the corresponding values obtained for the C_4_ sample. The C_3_ species with lower *S*_C/O_ and C_4_ species exhibit very similar oxygenase traits. These similarities suggest that the C_4_ plants sampled (*Panicum* and *Flaveria*) may have evolved from C_3_ with *S*_C/O_ well below the mean of the total C_3_ population. Notwithstanding the other preconditions (Gowik et al., 2011), if this restriction extends more generally to the *Poaceae* and *Asteraceae* families, it alone would have significantly limited the numbers of C_4_ species that could have evolved. The increased mean *V*_C_ observed in the C_4_ sample is consistent with adaptation in response to increased supply of CO_2_ to the enzyme following development of the CCM, decreasing the selection pressure to optimize the oxygenase reaction (*V*_O_, *K*_O_) and carboxylation (*K*_C_) traits.

### Photorespiration and the Homeostatic Maintenance of Chloroplast CO_2_ Levels in C_3_ Photosynthesis

Although the mitigation of photorespiration is seen as a pathway for improving crop yields, it is well recognized that under some conditions it is likely essential for healthy plant growth (Betti et al., 2016). Photorespiration can protect photosynthesis from light damage and help maintain cellular redox balance as well as plant immune responses (Voss et al., 2013). While the current evidence is largely circumstantial (Ratcliffe, 2018), another study suggests that photorespiration may not waste as much energy a first thought and enhances nitrate assimilation (Bloom and Lancaster, 2018). The scavenging of photorespiratory CO_2_ in plant cells helps maintain chloroplast *C* levels in C_3_ photosynthesis (Sage and Sage, 2009; Tholen and Zhu, 2011; Busch et al., 2013). The flow of chloroplast CO_2_ should be sufficient to occupy all available RuBisCO sites (Igamberdiev, 2015). When ambient CO_2_ decreases to lower than normal levels (as under extreme climatic conditions), CA may be unable to produce enough CO_2_ from the reservoir of bicarbonate to fuel RuBisCO, resulting in the underutilization of the energy produced by the light reactions, but this can be mitigated by the supply of photorespiratory CO_2_ (Igamberdiev, 2015). Moreover, the efficient operation of C_3_ photosynthesis may require that fluctuations in *O* and *C* be contained within certain limits (Roussel and Igamberdiev, 2011), which we expect would then tend to limit *v*_O/C_ (Equation 1). Based on these considerations, we might posit that *v*_O/C_ should also be maintained within certain limits. The availability of photorespiratory CO_2_ to chloroplasts supports a homeostatic mechanism that helps renormalizes *v*_O/C_ and CO_2_ levels by negative feedback of photorespiratory CO_2_ into chloroplasts in response to decreasing *C* levels.

Apart from substrate concentrations (*C* and *O*), the other factor that determines *v*_O/C_ is *S*_C/O_, which must then also be somehow constrained to keep *v*_O/C_ within certain limits. Loosely correlated changes in *S*_C_ and *S*_O_, which may result in an increase in one and a decrease in the other, have the potential to produce much larger variations in *S*_C/O_ than are observed (Figure 2D). The strong (*R*^2^>0.90) positive correlations between *S*_C_ and *S*_O_ produce tightly constrained *S*_C/O_ variability (Figure 2D), particularly within C_3_ genera (Figure 4A). An increase in *S*_C_ is accompanied by a proportionate increase in *S*_O_, facilitating the containment of *v*_O/C_ within the limits required for efficient photosynthesis. Such correlations between kinetic traits have usually been attributed to constraints inherent in RuBisCO’s catalytic mechanism (Tcherkez et al., 2006; Tcherkez, 2013, 2015; Flamholz et al., 2019), which we consider in detail below. However, another recent study suggests phylogenetic, rather than catalytic (or mechanistic), constraints have largely determine RuBisCO adaptation (Bouvier et al., 2021).

### RuBisCO Mechanistic Constraints

**Figure 6:**
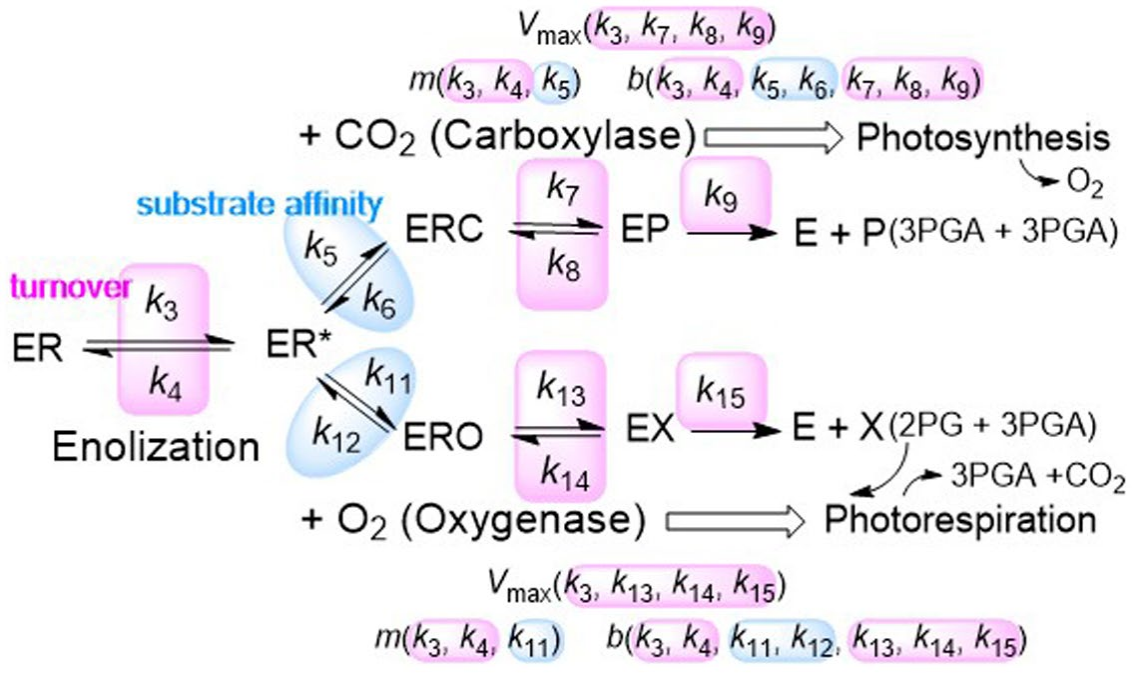
The kinetic mechanism of RuBisCO. RuBisCO processes the three substrates, ribulose-1,5-bisphosphate (RuBP), and CO_2_ or O_2_, the complete reactions taking place over several stages. RuBP (R) binds first, forming a complex (ER) with the activated form of the enzyme (E), followed by enolization of RuBP (ER^*^) which facilitates binding with the CO_2_ (C) or O_2_ (O) molecule to R^*^ forming the ERC or ERO enzyme-substrate complexes. The 6-carbon compound formed by the addition of carbon dioxide to RuBP breaks apart forming a product complex (EP) which dissociates into two molecules of the 3-carbon compound (P), 3-phosphoglyceric acid (3PGA). Oxygenation proceeds through analogous steps except that the dissociation products (X) are one 3PGA molecule and one of 2-phospho-glycolate (2PG) to be recycled into 3PGA by photorespiration, producing CO_2_ which can be made available for photosynthesis. The rate constants can be associated with turnover of product or affinity of CO_2_ or O_2_ substrate molecules for RuBP. The functional dependence of *V*_max_, *m* and *b* in *K*_M_ (Equation 3) on the elementary rate constants is indicated for each of the reactions. A derivation of the actual kinetic equations is given in the Supplementary Material.

A fundamental understanding of correlations between RuBisCO parameters requires consideration of its kinetic mechanism (Figure 6), together with a knowledge of the functional dependence of *V*_max_, *m* and *b* in Equation (3) on the rate constants (*k*_i_) for the elementary steps in both carboxylase and oxygenase reactions. As indicated in Figure 6, *V*_max_, *m* and *b* share a number of *k*_i_ associated with product turnover, while only *m* and *b* depend on the *k*_i_ that determine substrate affinity. Correlations between kinetic traits arising out of RuBisCOs mechanism must derive from correlations between the *k*_i_. However, the *k*_i_ are unknown quantities, difficult if not impossible to determine empirically with any certainty, and without simplifying assumptions (Tcherkez et al., 2006), complexity of the functional relationships hinders efforts to uncover such correlations (for details of the actual kinetic equations, see Cummins et al., 2018a). Nevertheless, several important observations can be made based on the results of the present analysis.

The absence of correlation between *S*_C_ and *V*_C_ in the C_3_ sample (Figure 1B) demonstrates that the *k*_i_ on which *V*_C_ depends are not correlated to that associated with CO_2_ affinity for enolized RuBP (*k*_5_). When *mV*_C_>>*b*, *S*_C_ converges asymptotically to the mean value for the sample. Compared to C_3_ *Limonium* and C_4_ plants, *S*_C_ converges very quickly to this mean value for the C_3_ sample (Table 1), and so the dependence of *K*_C_ on *b*, which depends on the decarboxylation rate (*k*_6_), may be neglected. This would suggest a lesser significance of *k*_6_ in C_3_ species, resolving to some extent the apparent differences of opinion on the issue of decarboxylation (Tcherkez et al., 2018; Cummins et al., 2019a). Nevertheless, the results suggest significant (*mV*_max_ ≈ *b*) decarboxylation in both C_3_ *Limonium* and C_4_ samples (Figure 1A) and deoxygenation (*k*_12_, i.e., breakdown of the Michaelis complex by dissociation of O_2_) in all three (Figure 1C).

Correlations between carboxylase and oxygenase reactions are not precluded. The carboxylase and oxygenase reactions are preceded by enolization of the bound RuBP required for activation of CO_2_ or O_2_ binding, which has been shown to co-limit *V*_max_ (Tcherkez et al., 2013). As a consequence of this co-limitation, the forward rate constant (*k*_3_) for the enolization of RuBP is a common factor of *V*_C_ and *V*_O_, which effectively couples the two reaction rates. However, the actual correlation observed between *V*_C_ and *V*_O_ is overall weak (Figure 2A) and variable between genera (Figure 5D), with only *Limonium* and *Aegilops* (both C_3_) exhibiting a moderate level of correlation. Consequently, these correlations are more likely due to adaptation, rather than by a tradeoff enforce by RuBisCO’s mechanism. In *Limonium*, the linear correlation between *V*_C_ and *V*_O_ maintains a more or less constant ratio *V*_C/O_ (in the range 2–3; Figure 2E), limiting variation in *S*_C/O_, and hence photorespiration, to mainly the ratio of *K*_M_s, i.e., *K*_O/C_ (Figure 2G).

In contrast, the positive correlations between *S*_C_ and *S*_O_ seem to establish a manifest constraint between carboxylase and oxygenase (Figures 2D, 4A) kinetic parameters. The correlation becomes stronger (as measured by *R*^2^) the more closely related the species; *R*^2^ values tend to be somewhat higher within genera (Figure 4A), than within the general C_3_, C_4_, or total plant sample (Figure 2D). The positive correlation seems to be preserved in mutants when the changes in kinetic parameters are relatively small (Genkov et al., 2010), while breaks for mutations that cause large perturbations to the kinetics, sometimes resulting in decreased *S*_C_ and increased *S*_O_, i.e., increasing photorespiration (Whitney et al., 1999). This tends to suggest some level of correlation between carboxylase and oxygenase *k*_i_, most likely with those associated with substrate affinity (Figure 6). Both substrates present similar electrostatic potentials to the RuBisCO active site (Kannappan and Gready, 2008), so that the binding of CO_2_ to enolized RuBP induces a redistribution of charge similar to that induced by O_2_ binding (Cummins et al., 2018b; Kannappan et al., 2019; Bathellier et al., 2020; Cummins and Gready, 2020) which will then interact similarly with the external (to the active site) electrostatic field, which is thought to be the primary driver in enzyme catalysis (Warshel et al., 2006; Fried and Boxer, 2017). Moreover, evolutionary changes in this electrostatic field have been linked to RuBisCO substrate specificity (Poudel et al., 2020) This electrostatic field would change slightly with sequence variation outside the highly conserved active site to produce the small free energy changes required to maintain the correlation between *S*_C_ and *S*_O_ when mutations occur. However, other types of biophysical constraints may limit the fitness of some mutations (Studer et al., 2014; Duraõ et al., 2015), and while superior traits are still being discovered (Davidi et al., 2020), the practical limits of RuBisCO’s kinetic variability remain unclear.

### Coevolution of RuBisCO, Photorespiration, and CCMs in Plants

The effect of abiotic environmental stress on RuBisCO in C_3_ plants may be succinctly rationalized in terms of the ratios of *V*_max_, *V*_C/O_=*V*_C_/*V*_O_, and *K*_M_s, *K*_O/C_=*K*_O_/*K*_C_ (*V*_C/O_ vs. *K*_O/C_ in Figure 2H). Both the carboxylase and oxygenase reactions produce 3PGA (Figure 6), although RuBisCO processes the primary substrate (CO_2_) more efficiently than O_2_ due to superior kinetic traits (*V*_max_, *K*_M_) and the fact that one of the oxygenase products (2PG) has to be reprocessed into 3PGA by photorespiration, albeit costing a certain amount of additional energy and carbon. On the other hand, the additional CO_2_ produced as a byproduct in photorespiration may be reutilized in photosynthesis if captured by chloroplasts. Thus *V*_C/O_ seems to strike a practical balance between photosynthesis and photorespiration as a measure of 3PGA production efficiency; the higher *V*_C/O_ the more energy efficiently 3PGA is produced. According to the familiar form of the classical Michaelis-Menten (MM) equation (Michaelis and Menten, 1913; Briggs and Haldane, 1925; Michaelis et al., 2011),

(8) $$v=\frac{V_{\max}\left[S\right]}{K_{M}+\left[S\right]}$$

when the reaction rate (*v*) reaches half of *V*_max_, *K*_M_ is equivalent to the concentration of substrate, [S]. Consequently, for a given *V*_max_, a substrate with a lower value of *K*_M_ saturates the enzyme with a smaller concentration of substrate. Actually (*in vivo*), the rate of carbon assimilation in plants typically deviates from the classical MM curve (Equation 8) reaching only about 50% of *V*_C_ (Laisk, 1985; Laisk and Oja, 1998; Ruuska et al., 1998) due to some other limiting factors (Igamberdiev, 2015). To reach saturation, lower values of *K*_C_ require less CO_2_, and higher values of *K*_O_ more O_2_. In terms of the RuBisCO kinetic mechanism (Figure 6), lower *K*_C_ can be best achieved by increasing CO_2_ affinity (*k*_5_/*k*_6_), and higher *K*_O_ by decreasing O_2_ affinity (*k*_11_/*k*_12_) for the enolizied form of RuBP.

**Figure 7:**
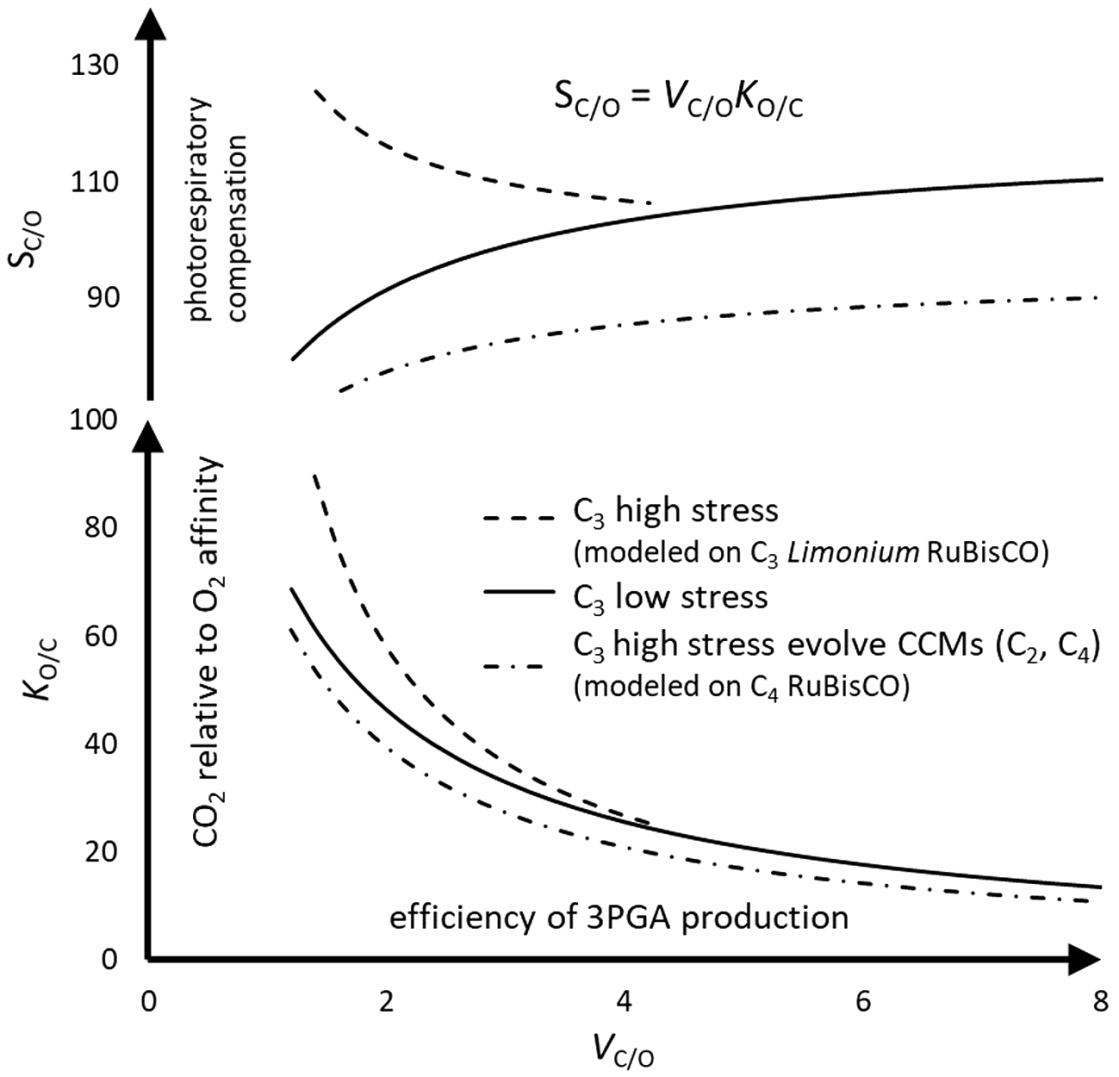
The effect of abiotic environmental stress on RuBisCO kinetics in C_3_ plants. The trend lines (Figure 2H) in relative specificity, *S*_C/O_, are obtained by the product of *V*_C/O_=*V*_C_/*V*_O_, a measure of 3PGA product turnover efficiency, and *K*_O/C_=*K*_O_/*K*_C_, a measure of CO_2_ relative to O_2_ affinity for enolizied RuBP. The efficiency of C_3_ photosynthesis also relies on the constant supply of CO_2_ to RuBisCO, which photorespiration can help maintain. Abiotic stress factors increase the photorespiration relative to photosynthesis (Equation 1). Most C_3_ species, with more or less average *S*_C/O_, are situated in usually low stress (or temperate) habitats; however, some level of photorespiratory CO_2_ may be necessary to maintain photosynthesis during short periods of increased stress (Igamberdiev, 2015). Some C_3_ plants can leverage off the additional photorespiratory CO_2_ produced by their much lower than average *S*_C/O_ in high stress environments (C_2_ photosynthesis). Some of these C_2_ plants may go on to evolve fully developed carbon concentrating mechanisms (C_4_ photosynthesis). Alternatively, the much higher than average *S*_C/O_ in other C_3_ plants, while mitigating photorespiration, may compensate for the carbon restriction (reduced levels of chloroplast CO_2_) associated with high stress environments by gains in CO_2_ relative to O_2_ affinity.

The curvature of the predicted trend lines in Figure 7 is largely determined by the explicit linear dependence of *K*_M_ on *V*_max_ (Equation 3); increasing *V*_C/O_ produces the monotonic decrease in *K*_O/C_. There is no obvious explanation for the divergence of *K*_O/C_, and hence *S*_C/O_, curves between C_3_ *Limonium* and the mainstream C_3_ species at lower *V*_C/O_ as arising from mechanistic constrains imposed by the enzyme. Alternatively, it is posited that the observed trends arise from adaptation of RuBisCO in response to changes in chloroplast carbon (*C*) levels according to the prevailing environmental conditions. Under more temperate conditions (applicable to most of the C_3_ sample), in species with less than the mean *V*_C/O_ of about three (Table 1), *C* can be supplemented by increased photorespiration (decreasing *S*_C/O_). Increasing *S*_C/O_ reduces photorespiration and hence the maintenance of *C*, requiring increased *K*_O/C_ (higher CO_2_ relative to O_2_ affinity) to maintain sustainable levels of photosynthesis under high stress at the lower end of *V*_C/O_. In fact, a unique study provides some empirical evidence that those (*Limonium*) species with higher *S*_C/O_ are associated with reduced *C* (Galmés et al., 2014).

As *V*_C/O_ increases from its mean toward the maximum value, the requirement for additional carbon appears to diminish; photorespiratory CO_2_ declines with a drift toward increased *S*_C/O_. Thus, there is a positive correlation between *V*_C/O_ and photorespiratory *C*, which is also clearly apparent in C_4_ evolution where the establishment of increased *C* levels (by the CCM) is followed by optimization of *V*_C_. Increased throughput of product can only be maintained by increased supply of CO_2_ substrate, necessitating the adaptation of RuBisCO kinetic traits. Most of the C_3_ sample is tightly clustered about the mean *V*_C_ or *V*_C/O_ (Figures 2C,E). If *V*_C/O_ falls much below the mean, *C* requires supplementation depending on environmental conditions, either by increased photorespiration (low stress) or by increased CO_2_ relative to O_2_ affinity (high stress), necessitating RuBisCO accommodate an expansive range of *S*_C/O_. As discussed, C_4_ plants have likely evolved only from C_3_ with below average *S*_C/O_, and this appears to be supported by the parallel trends in C_3_ and C_4_ illustrated in Figure 7. C_3_ species with the minimal values of *S*_C/O_ (lower values for *K*_O/C_) produce additional photorespiratory CO_2_ under high stress conditions, as required for the evolution of C_2_ and C_4_ photosynthesis. The CCM maintains consistently higher *C* levels, regardless of stress factors, reducing the pressure on RuBisCO to adapt, so that its kinetics have remained, except perhaps for a tendency toward higher *V*_C_, relatively unchanged by evolution.

## Conclusion

Analysis of the RuBisCO kinetic data presented here suggests that the evolution of kinetic parameters in higher plants, rather than being highly constrained or subjected to tradeoffs imposed by the enzyme’s kinetic mechanism, has adapted to variations in photorespiration as part the homeostatic maintenance of a constant CO_2_ supply to the enzyme under disparate environmental conditions. The positive correlations observed between *S*_C_ and *S*_O_, particularly between phylogenetically related species, reflect similarities in the physical binding properties of the two substrates CO_2_ and O_2_ to RuBP, which serve to contain *S*_C/O_ within the limits required for maintaining balance between photosynthesis and photorespiration in the regulation of carbon flux when mutations occur. Significantly, the limitation on product turnover (*V*_C_) is the extent to which RuBisCO kinetics can adapt to the availability of carbon. Apparent tradeoffs between turnover and specificity are not symptomatic of an inefficient enzyme, but reflects the necessary adaptation of a flexible one to the changing levels of accessible CO_2_ as a consequence of changes in abiotic environmental conditions.

Over the past 30ma, the evolution of C_4_ photosynthesis has dramatically reduced photorespiration in a relatively small number (a few percent) of plant species by maintaining high levels of chloroplast CO_2_, although somewhat paradoxically high levels of photorespiration were instrumental at the beginning the evolutionary process. The C_4_ RuBisCOs in the *Panicum* and *Flaveria* samples do not exhibit the “average” C_3_ kinetics, but inherit traits largely unchanged from a small proportion of the ancestral C_3_ population (i.e., those with much lower than the mean *S*_C/O_ and, therefore, increased photorespiration). Nevertheless, differences in leaf anatomy and biochemistry indicate that a C_4_ plant is not simply a C_3_ with an attached CCM, and prodigious efforts to artificially introduce CCMs into commercial C_3_ crops are ongoing but have yet to bear fruit. Recent modeling suggests that achieving C_2_ photosynthesis in rice may be a more realistic goal (Bellasio and Farquhar, 2019).

While this research continues, however, are there any prospects for reengineering C_3_ RuBisCOs with the objective of improving its kinetic traits? Despite decades of research, while some progress has been made through directed evolution, a demonstrably better RuBisCO for agriculture also remains elusive (Zhou and Whitney, 2019). What is meant by a “better” RuBisCO needs to be carefully defined, and the traits to be improved should at least, if not necessarily improve yield under the prevailing settings, increase the fitness of a species to survive the expected increases in the frequency and severity of hot and dry weather events over the coming decades. In this regard, C_4_ plants demonstrate the importance of a secure supply of carbon under such climatic conditions, and the evolution of increases in product turnover (*V*_C_), although perhaps modest compared to *V*_C_ found in other taxa (Davidi et al., 2020), may well only have been maintained by the increased availability of carbon provided by the CCM. C_3_ plants with higher *S*_C/O_ suffer from the concomitant reduction in the contributions to chloroplast CO_2_ levels that would otherwise had been made by photorespiration. In these cases, increasing the affinity of CO_2_ relative to O_2_ for RuBP makes more efficient uptake of the meager supply of carbon under high stress environmental conditions. In the absence of a functional CCM, the reengineering of RuBisCOs with increased *S*_C/O_, if possible, may help to futureproof C_3_ crops in a rapidly changing climate.

## Data Availability Statement

Publicly available data sets were analyzed in this study. This data can be found at: https://pubs.acs.org/doi/abs/10.1021/acs.biochem.9b00237/supplfile/bi9b00237si005.xlsx.

## Author Contributions

The author confirms being the sole contributor of this work and has approved it for publication.

## Conflict of Interest

The author declares that the research was conducted in the absence of any commercial or financial relationships that could be construed as a potential conflict of interest.

## Publisher’s Note

All claims expressed in this article are solely those of the authors and do not necessarily represent those of their affiliated organizations, or those of the publisher, the editors and the reviewers. Any product that may be evaluated in this article, or claim that may be made by its manufacturer, is not guaranteed or endorsed by the publisher.

## Supplementary Material

The Supplementary Material for this article can be found online at: https://www.frontiersin.org/articles/10.3389/fpls.2021.662425/full#supplementary-material, www.real-statistics.com, www.c4rice.com

## References

[ref1] Andralojc P. J. Carmo-Silva E. Degen G. E. Parry M. A. J. (2018). Increasing metabolic potential: C-fixation. Essays Biochem. 62, 109–118. 10.1042/EBC20170014, PMID: 29653967

[ref2] Atkinson N. Mao Y. Chan K. X. McCormick A. J. (2020). Condensation of Rubisco into a proto-pyrenoid in higher plant chloroplasts. Nat. Commun. 11:6303. 10.1038/s41467-020-20132-0, PMID: 33298923 PMC7726157

[ref3] Bar-Even A. Noor E. Savir Y. Liebermeister W. Davidi D. Tawfik D. S.. (2011). The moderately efficient enzyme: evolutionary and physicochemical trends shaping enzyme parameters. Biochemistry 50, 4402–4410. 10.1021/bi2002289, PMID: 21506553

[ref4] Bathellier C. Li-Juan Yu L.-J. Farquhar G. D. Michelle L. Coote M. L. Lorimer G. H.. (2020). Ribulose 1,5-bisphosphate carboxylase/oxygenase activates O_2_ by electron transfer. Proc. Natl. Acad. Sci. 10.1073/pnas.2008824117, PMID: 32934141 PMC7533879

[ref5] Bellasio C. Farquhar G. (2019). A leaf-level biochemical model simulating the introduction of C_2_ and C_4_ photosynthesis in C3rice: gains, losses and metabolitefluxes. New Phytol. 223, 150–166. 10.1111/nph.15787, PMID: 30859576

[ref6] Betti M. Bauwe H. Busch F. A. Fernie A. R. Keech O. Levey M.. (2016). Manipulating photorespiration to increase plant productivity: recent advances and perspectives for crop improvement. J. Exp. Bot. 67, 2977–2988. 10.1093/jxb/erw076, PMID: 26951371

[ref7] Bloom A. J. Lancaster K. M. (2018). Manganese binding to Rubisco could drive a photorespiratory pathway that increases the energy efficiency of photosynthesis. Nat. Plants 4, 414–422. 10.1038/s41477-018-0191-0, PMID: 29967515

[ref8] Blount Z. D. Lenski R. E. Losos J. B. (2018). Contingency and determinism in evolution: replaying life’s tape. Science 362:eaam5979. 10.1126/science.aam5979, PMID: 30409860

[ref9] Bouvier J. W. Emms D. M. Rhodes T. Nielsen J. R. Bolton J. S. Eddershaw A.. (2021). Rubisco adaptation is more limited by phylogenetic constraint than by catalytic trade-off. Mol. Biol. Evol. 25, 2880–2896. 10.1093/molbev/msab079, PMID: 33739416 PMC8233502

[ref10] Bräutigam A. Gowik U. (2016). Photorespiration connects C_3_ and C_4_ photosynthesis. J. Exp. Bot. 67, 2953–2962. 10.1093/jxb/erw056, PMID: 26912798

[ref11] Briggs G. E. Haldane J. B. S. (1925). A note on the kinetics of enzyme action. Biochem. J. 19, 338–339. 10.1042/bj0190338, PMID: 16743508 PMC1259181

[ref12] Busch F. A. Sage T. L. Cousins A. B. Sage R. F. (2013). C_3_ plants enhance rates of photosynthesis by reassimilating photorespired and respired CO_2_. Plant Cell Environ. 36, 200–212. 10.1111/j.1365-3040.2012.02567.x, PMID: 22734462

[ref13] Christin P. A. Osborne C. P. Chatelet D. S. Columbus J. T. Besnard G. Hodkinson T. R.. (2013). Anatomical enablers and the evolution of C_4_ photosynthesis in grasses. Proc. Natl. Acad. Sci. U. S. A. 110, 1381–1386. 10.1073/pnas.1216777110, PMID: 23267116 PMC3557070

[ref14] Cleland W. W. Andrews T. J. Gutteridge S. Hartmann F. C. Lorimer G. H. (1998). Mechanism of Rubisco: the carbamate as general base. Chem. Rev. 98, 549–562. 10.1021/cr970010r, PMID: 11848907

[ref15] Cumming G. (2012). Understanding the New Statistics: Effect Sizes, Confidence Interval and Meta Analysis. Taylor and Francis, New York, 53–117.

[ref16] Cummins P. L. Kannappan B. Gready J. E. (2018a). Directions for optimization of photosynthetic carbon fixation: Rubisco's efficiency may not be so constrained after all. Front. Plant Sci. 9:183. 10.3389/fpls.2018.00183, PMID: 29545812 PMC5838012

[ref17] Cummins P. L. Kannappan B. Gready J. E. (2018b). Revised mechanism of carboxylation of ribulose-1,5-biphosphate by Rubisco from large scale quantum chemical calculations. J. Comput. Chem. 39, 1656–1665. 10.1002/jcc.25343, PMID: 29756365

[ref18] Cummins P. L. Kannappan B. Gready J. E. (2019a). Response: commentary: directions for optimization of photosynthetic carbon fixation: Rubisco’s efficiency may not be so constrained after all. Front. Plant Sci. 10:1426. 10.3389/fpls.2019.01426, PMID: 31824523 PMC6884029

[ref19] Cummins P. L. Kannappan B. Gready J. E. (2019b). Ab initio molecular dynamics simulation and energetics of the ribulose-1,5-biphosphate carboxylation reaction catalyzed by Rubisco: toward elucidating the stereospecific protonation mechanism. J. Phys. Chem. B 123, 2679–2686. 10.1021/acs.jpcb.8b12088, PMID: 30807177

[ref20] Cummins P. L. Gready J. E. (2020). Kohn-Sham density functional calculations reveal proton wires in the enolization and carboxylase reactions catalyzed by Rubisco. J. Phys. Chem. B 124, 3015–3026. 10.1021/acs.jpcb.0c01169, PMID: 32208706

[ref21] Davidi D. Shamshoum M. Guo Z. Bar-On Y. M. Prywes N. Oz A.. (2020). Highly active Rubiscos discovered by systematic interrogation of natural sequence diversity. EMBO J. 39:e104081. 10.15252/embj.2019104081, PMID: 32500941 PMC7507306

[ref22] Duraõ P. Aigner H. Nagy P. Mueller-Cajar O. Hartl F. U. Hayer-Hartl M. (2015). Opposing effects of folding and assembly chaperones on evolvability of 694 Rubisco. Nat. Chem. Biol. 11, 148–155. 10.1038/nchembio.1715, PMID: 25558973

[ref23] Erb T. J. Zarzycki J. (2018). A short history of RubisCO: the rise and fall (?) of nature's predominant CO_2_ fixing enzyme. Curr. Opin. Biotechnol. 49, 100–107. 10.1016/j.copbio.2017.07.017, PMID: 28843191 PMC7610757

[ref24] Fernie A. R. Bachem C. W. B. Helariutta Y. Neuhaus E. Prat S. Ruan Y.-L.. (2020). Synchronization of developmental, molecular and metabolic aspects of source–sink interactions. Nat. Plants 6, 55–66. 10.1038/s41477-020-0590-x, PMID: 32042154

[ref25] Flamholz A. I. Prywes N. Moran U. Davidi D. Bar-On Y. M. Oltrogge L. M.. (2019). Revisiting tradeoffs in Rubisco kinetic parameters. Biochemistry 58, 3365–3376. 10.1021/acs.biochem.9b00237, PMID: 31259528 PMC6686151

[ref26] Fried S. D. Boxer S. G. (2017). Electric fields and enzyme catalysis. Annu. Rev. Biochem. 86, 387–415. 10.1146/annurev-biochem-061516-044432, PMID: 28375745 PMC5600505

[ref27] Gomez-Fernandez B. J. Garcia-Ruiz E. Martin-Diaz J. Gomez de Santos P. Santos-Moriano P. Plou F. J.. (2018). Directed -in vitro- evolution of precambrian and extant Rubiscos. Sci. Rep. 8:5532. 10.1038/s41598-018-23869-3, PMID: 29615759 PMC5883036

[ref28] Galmés J. Flexas J. Keys A. J. Cifre J. Mitchell R. A. C. Madgwick P. J.. (2005). Rubisco specificity factor tends to be larger in plant species from drier habitats and in species with persistent leaves. Plant Cell Environ. 28, 571–579. 10.1111/j.1365-3040.2005.01300.x

[ref29] Galmés J. Andralojc P. J. Kapralov M. V. Flexas J. Keys A. J. Molins A.. (2014). Environmentally driven evolution of Rubisco and improved photosynthesis and growth within the C_3_ genus *Limonium* (Plumbaginaceae). New Phytol. 203, 989–999. 10.1111/nph.12858, PMID: 24861241

[ref30] Genkov T. Meyer M. Griffiths H. Spreitzer R. J. (2010). Functional hybrid rubisco enzymes with plant small subunits and algal large subunits: engineered rbcS cDNA for expression in chlamydomonas. J. Biol. Chem. 285, 19833–19841. 10.1074/jbc.M110.124230, PMID: 20424165 PMC2888394

[ref31] Goudet M. Orr D. Melkonian M. Muller K. Meyer M. Carmo-Silva E.. (2020). Rubisco and carbon concentration mechanism (CCM) co-evolution across Chlorophytes and Streptophytes. New Phytol. 227, 810–823. 10.1111/nph.16577, PMID: 32249430

[ref32] Gowik U. Bräutigam A. Weber K. L. Weber A. P. M. Westhoff P. (2011). Evolution of C_4_ photosynthesis in the genus *Flaveria*: how many and which genes does it take to make C_4_? Plant Cell 23, 2087–2105. 10.1105/tpc.111.086264, PMID: 21705644 PMC3160039

[ref33] Hanson D. T. (2016). Breaking the rules of Rubisco catalysis. J. Exp. Bot. 67, 3180–3182. 10.1093/jxb/erw197, PMID: 27241490 PMC4892744

[ref34] Igamberdiev A. U. (2015). Control of Rubisco function via homeostatic equilibration of CO_2_ supply. Front. Plant Sci. 6:106. 10.3389/fpls.2015.00106, PMID: 25767475 PMC4341507

[ref35] Igamberdiev A. U. Roussel M. R. (2012). Feedforward non-Michaelis–Menten mechanism for CO_2_ uptake by Rubisco: contribution of carbonic anhydrases and photorespiration to optimization of photosynthetic carbon assimilation. Biosystems 107, 158–166. 10.1016/j.biosystems.2011.11.008, PMID: 22154946

[ref36] Iñiguez C. Capó-Bauçà S. Niinemets Ü. Stoll H. Aguiló-Nicolau P. Galmés J. (2020). Evolutionary trends in Rubisco kinetics and their co-evolution with CO_2_ concentrating mechanisms. Plant J. 101, 897–918. 10.1111/tpj.14643, PMID: 31820505

[ref37] Jordan D. B. Ogren W. L. (1984). The CO_2_/O_2_ specificity of ribulose 1,5-bisphosphate carboxylase oxygenase: dependence on ribulosebisphosphate concentration, pH and temperature. Planta 161, 308–313. 10.1007/BF00398720, PMID: 24253719

[ref38] Jeske L. Placzek S. Schomburg I. Chang A. Schomburg D. (2019). BRENDA in 2019: a European ELIXIR core data resource. Nucleic Acids Res. 47, D542–D549. 10.1093/nar/gky1048, PMID: 30395242 PMC6323942

[ref39] Jurić I. Hibberd J. M. Blatt M. Burroughs N. J. (2019). Computational modelling predicts substantial carbon assimilation gains for C_3_ plants with a single-celled C_4_ biochemical pump. PLoS Comput. Biol. 15:e1007373. 10.1371/journal.pcbi.1007373, PMID: 31568503 PMC6786660

[ref40] Kannappan B. Cummins P. L. Gready J. E. (2019). Mechanism of oxygenase pathway reactions catalyzed by Rubisco from large scale Kohn-Sham density functional calculations. J. Phys. Chem. B 123, 2833–2843. 10.1021/acs.jpcb.9b00518, PMID: 30845802

[ref41] Kannappan B. Gready J. E. (2008). Redefinition of rubisco carboxylase reaction reveals origin of water for hydration and new roles for active-site residues. J. Am. Chem. Soc. 130, 15063–15080. 10.1021/ja803464a, PMID: 18855361

[ref42] Kapralov M. V. Kubien D. S. Andersson I. Filatov D. A. (2011). Changes in Rubisco kinetics during the evolution of C_4_ photosynthesis in *Flaveria* (Asteraceae) are associated with positive selection on genes encoding the enzyme. Mol. Biol. Evol. 28, 1491–1503. 10.1093/molbev/msq335, PMID: 21172830

[ref43] Kubien D. S. Whitney S. M. Moore P. V. Jesson L. K. (2008). The biochemistry of Rubisco in *Flaveria*. J. Exp. Bot. 59, 1767–1777. 10.1093/jxb/erm283, PMID: 18227079

[ref44] Kubis A. Bar-Even A. (2019). Synthetic biology approaches for improving photosynthesis. J. Exp. Bot. 70, 1425–1433. 10.1093/jxb/erz029, PMID: 30715460 PMC6432428

[ref45] Laisk A. (1985). “Kinetics of photosynthetic CO_2_ uptake in C3 plants,” in Kinetics of Photosynthetic Carbon Metabolism. eds. Viil J. Grishina G. Laisk A. (Tallinn: Valgus Press), 21–34.

[ref46] Laisk A. Oja V. (1998). Dynamics of Leaf Photosynthesis: Rapid Response Measurements and Their Interpretations. Melbourne: CSIRO Publishing.

[ref47] Laing W. A. Ogren W. L. Hageman R. H. (1974). Regulation of soybean net photosynthetic CO_2_ fixation by the interaction of CO_2_, O_2_ and ribulose 1,5-bisphosphate carboxylase. Plant Physiol. 54, 678–685. 10.1104/pp.54.5.678, PMID: 16658951 PMC366581

[ref48] Lawson T. Flexus J. (2020). Fuelling life: recent advances in photosynthesis research: editorial. Plant J. 101, 753–755. 10.1111/tpj.14698, PMID: 32100942

[ref49] Lin M. T. Stone W. D. Chaudhari V. Hanson M. R. (2020). Small subunits can determine enzyme kinetics of tobacco Rubisco expressed in *Escherichia coli*. Nat. Plants 6, 1289–1299. 10.1038/s41477-020-00761-5, PMID: 32929197

[ref50] Mallmann J. Heckmann D. Bräutigam A. Lercher M. J. Weber A. P. Westhoff P.. (2014). The role of photorespiration during the evolution of C_4_ photosynthesis in the genus *Flaveria*. ELife 3:e02478. 10.7554/eLife.02478, PMID: 24935935 PMC4103682

[ref51] McGrath J. M. Long S. P. (2014). Can the cyanobacterial carbon-concentrating mechanism increase photosynthesis in crop species? Plant Physiol. 164, 2247–2261. 10.1104/pp.113.232611, PMID: 24550242 PMC3982776

[ref52] McKown A. D. Moncalvo J. M. Dengler N. G. (2005). Phylogeny of *Flaveria* (Asteraceae) and inference of C_4_ photosynthesis evolution. Am. J. Bot. 92, 1911–1928. 10.3732/ajb.92.11.1911, PMID: 21646108

[ref53] Michaelis L. Menten M. (1913). Die kinetik der invertinwirkung. Biochem. Z. 49, 333–369.

[ref54] Michaelis L. Menten M. L. Johnson K. A. Goody R. S. (2011). The original Michaelis constant: translation of the 1913 Michaelis-Menten paper. Biochemistry 50, 8264–8269. 10.1021/bi201284u, PMID: 21888353 PMC3381512

[ref55] Mundry R. (2014). “Statistical Issues and Assumptions of Phylogenetic Generalized Least Squares,” in Modern Phylogenetic Comparative Methods and Their Application in Evolutionary Biology. ed. Garamszegi L. Z. (Springer-Verlag Berlin Heidelberg), 131–153.

[ref56] Niinemets U. Berry J. A. von Caemmerer S. Ort D. R. Parry M. A. J. Poorter H. (2017). Photosynthesis: ancient, essential, complex, diverse … and in need of improvement in a changing world. New Phytol. 213, 43–47. 10.1111/nph.14307, PMID: 27891642

[ref57] Poudel S. Pike D. H. Raanan H. Mancini J. A. Nanda V. Rickaby R. E. M.. (2020). Biophysical analysis of the structural evolution of substrate specificity in Rubisco. Proc. Natl. Acad. Sci. 117, 30451–30457. 10.1073/pnas.2018939117, PMID: 33199597 PMC7720200

[ref58] Ratcliffe G. (2018). Faculty opinions recommendation of Bloom A.J. and Lancaster K.M., *Nat. Plants*. Fac. Opin. 4, 414–422. 10.3410/f.733575302.793548683 29967515

[ref59] Riazunnisa K. Padmavathi L. Bauwe H. Raghavendra A. S. (2006). Markedly low requirement of added CO_2_ for photosynthesis by mesophyll protoplasts of pea (*Pisum sativum*): possible roles of photorespiratory CO_2_ and carbonic anhydrase. Physiol. Plant. 128, 763–772. 10.1111/j.1399-3054.2006.00803.x

[ref60] Roussel M. R. Igamberdiev A. U. (2011). Dynamics and mechanisms of oscillatory photosynthesis. Biosystems 103, 230–238. 10.1016/j.biosystems.2010.07.020, PMID: 20739004

[ref61] Ruuska S. A. Andrews T. J. Badger M. R. Hudson G. S. Laisk A. Price G. D.. (1998). The interplay between limiting processes in C_3_ photosynthesis studied by rapid response gas exchange using transgenic tobacco impaired in photosynthesis. Aust. J. Plant Physiol. 25, 859–870. 10.1071/PP98079

[ref62] Sage R. F. (2004). The evolution of C_4_ photosynthesis. New Phytol. 161, 341–370. 10.1111/j.1469-8137.2004.00974.x, PMID: 33873498

[ref63] Sage R. F. (2013). Photorespiratory compensation: a driver for biological diversity. Plant Biol. 15, 624–638. 10.1111/plb.12024, PMID: 23656429

[ref64] Sage R. F. (2016). A portrait of the C_4_ photosynthetic family on the 50th anniversary of its discovery: species number, evolutionary lineages, and Hall of Fame. J. Exp. Bot. 67, 4039–4056. 10.1093/jxb/erw156, PMID: 27053721

[ref65] Sage T. L. Sage R. F. (2009). The functional anatomy of rice leaves: implications for refixation of photorespiratory CO_2_ and efforts to engineer C_4_ photosynthesis into rice. Plant Cell Physiol. 50, 756–772. 10.1093/pcp/pcp033, PMID: 19246459

[ref66] Sage T. L. Busch F. A. Johnson D. C. Friesen P. C. Stinson C. R. Stata M.. (2013). Initial events during the evolution of C_4_ photosynthesis in C_3_ species of *Flaveria*. Plant Physiol. 163, 1266–1276. 10.1104/pp.113.221119, PMID: 24064930 PMC3813649

[ref67] Sage R. F. Christin P.-A. Edwards E. J. (2011). The C_4_ plant lineages of planet Earth. J. Exp. Bot. 62, 3155–3169. 10.1093/jxb/err048, PMID: 21414957

[ref68] Sage R. F. Monson R. K. Ehleringer J. R. Adachi S. Pearcy R. W. (2018). Some like it hot: the physiological ecology of C_4_ plant evolution. Oecologia 187, 941–966. 10.1007/s00442-018-4191-6, PMID: 29955992

[ref69] Sage R. F. Sage T. L. Kocacinar F. (2012). Photorespiration and the evolution of C_4_ photosynthesis. Annu. Rev. Plant Biol. 63, 19–47. 10.1146/annurev-arplant-042811-105511, PMID: 22404472

[ref70] Savir Y. Noor E. Milo R. Tlusty T. (2010). Cross-species analysis traces adaptation of Rubisco toward optimality in a low-dimensional landscape. Proc. Natl. Acad. Sci. U. S. A. 107, 3475–3480. 10.1073/pnas.0911663107, PMID: 20142476 PMC2840432

[ref71] Schulze S. Mallmann J. Burscheidt J. Koczor M. Streubel M. Bauwe H.. (2013). Evolution of C_4_ photosynthesis in the genus *Flaveria*: establishment of a photorespiratory CO_2_ pump. Plant Cell 25, 2522–2535. 10.1105/tpc.113.114520, PMID: 23847152 PMC3753380

[ref72] Schüssler C. Freitag H. Koteyeva N. Schmidt D. Edwards G. Vosnesenskya E.. (2017). Molecular phylogeny and forms of photosynthesis in tribe Salsoleae (Chenopodiaceae). J. Exp. Bot. 68, 207–223. 10.1093/jxb/erw432, PMID: 28003310 PMC5853613

[ref73] Sharwood R. E. Ghannoum O. Kapralov M. V. Gunn L. H. Whitney S. M. (2016). Temperature responses of Rubisco from *Paniceae* grasses provide opportunities for improving C_3_ photosynthesis. Nat. Plants 2:16186. 10.1038/nplants.2016.186, PMID: 27892943

[ref74] Studer R. A. Christin P. Williams M. A. Orengo C. A. (2014). Stability activity tradeoffs constrain the adaptive evolution of RubisCO. Proc. Natl. Acad. Sci. U. S. A. 111, 2223–2228. 10.1073/pnas.1310811111, PMID: 24469821 PMC3926066

[ref75] Tcherkez G. G. Farquhar G. D. Andrews T. J. (2006). Despite slow catalysis and confused substrate specificity, all ribulose bisphosphate carboxylases may be nearly perfectly optimized. Proc. Natl. Acad. Sci. U. S. A. 103, 7246–7251. 10.1073/pnas.0600605103, PMID: 16641091 PMC1464328

[ref76] Tcherkez G. G. B. Bathellier C. Stuart-Williams H. Whitney S. Gout E. Bligny R.. (2013). D_2_O solvent isotope effects suggest uniform energy barriers in ribulose1,5-bisphosphate carboxylase/oxygenase catalysis. Biochemistry 52, 869–877. 10.1021/bi300933u, PMID: 23301499

[ref77] Tcherkez G. (2013). Modelling the reaction mechanism of ribulose-1,5-bisphosphate carboxylase/oxygenase and consequences for kinetic parameters. Plant Cell Environ. 36, 1586–1596. 10.1111/pce.12066, PMID: 23305122

[ref78] Tcherkez G. (2015). The mechanism of rubisco-catalyzed oxygenation. Plant Cell Environ. 39, 983–1596. 10.1111/pce.12629 26286702

[ref79] Tcherkez G. G. Bathellier C. Farquhar G. D. Lorimer G. H. (2018). Commentary: directions for optimization of photosynthetic carbon fixation: Rubisco’s efficiency may not be so constrained after all. Front. Plant Sci. 9:183. 10.3389/fpls.2018.00929, PMID: 29997647 PMC6030380

[ref80] Tholen D. Zhu X.-G. (2011). The mechanistic basis of internal conductance: a theoretical analysis of mesophyll cell photosynthesis and CO_2_ diffusion. Plant Physiol. 156, 90–105. 10.1104/pp.111.172346, PMID: 21441385 PMC3091052

[ref81] Thompson M. Gamage D. Hirotsu N. Martin A. Seneweera S. (2017). Effects of elevated carbon dioxide on photosynthesis and carbon partitioning: a perspective on root sugar sensing and hormonal crosstalk. Front. Physiol. 8:578. 10.3389/fphys.2017.00578, PMID: 28848452 PMC5550704

[ref82] Voss I. Sunil B. Scheibe R. Raghavendra A. S. (2013). Emerging concept for the role of photorespiration as an important part of abiotic stress response. Plant Biol. 15, 713–722. 10.1111/j.1438-8677.2012.00710.x, PMID: 23452019

[ref83] Warshel A. Sharma P. K. Kato M. Xiang Y. Liu H. Olsson M. H. M. (2006). Electrostatic basis for enzyme catalysis. Chem. Rev. 106, 3210–3235. 10.1021/cr0503106, PMID: 16895325

[ref84] Wilson R. H. Martin-Avila E. Conlan C. Whitney S. M. (2018). An improved *Escherichia coli* screen for Rubisco identifies a protein–protein interface that can enhance CO_2_-fixation kinetics. J. Biol. Chem. 293, 18–27. 10.1074/jbc.M117.810861, PMID: 28986448 PMC5766918

[ref85] Whitney S. M. von Caemmerer S. Hudson G. S. Andrews T. J. (1999). Directed mutation of the Rubisco large subunit of tobacco influences photorespiration and growth. Plant Physiol. 121, 579–588. 10.1104/pp.121.2.579, PMID: 10517850 PMC59421

[ref86] Whitney S. M. Houtz R. L. Alonso H. (2011). Advancing our understanding and capacity to engineer nature's CO2-sequestering enzyme, Rubisco. Plant Physiol. 155, 27–35. 10.1104/pp.110.164814, PMID: 20974895 PMC3075749

[ref87] Xu Z. Jiang Y. Jia B. Zhou G. (2016). Elevated-CO_2_ response of stomata and its dependence on environmental factors. Front. Plant Sci. 7:657. 10.3389/fpls.2016.00657, PMID: 27242858 PMC4865672

[ref88] Zaiontz C. (2020). Real Statistics Resource Pack software (Release 7.2).

[ref89] Zhou Y. Whitney S. (2019). Directed evolution of an improved RuBisCO; in vitro analyses to decipher fact from fiction. Int. J. Mol. Sci. 20, 5019–5039. 10.3390/ijms20205019, PMID: 31658746 PMC6834295

