# Supplementary material for: The Coevolution of RuBisCO, Photorespiration, and Carbon Concentrating Mechanisms in Higher Plants
Source: Front Plant Sci. 2021 Sep 1;12:662425. doi: 10.3389/fpls.2021.662425 (PMC8440988; doi:10.3389/fpls.2021.662425)
Supplement: Supplementary file 1 [file Data_Sheet_1.PDF]

Supplementary Material for: The Co-evolution of RuBisCO, Photorespiration and Carbon Concentrating Mechanisms in Higher Plants

(**Peter L. Cummins,** Department of Genome Sciences, John Curtin School of Medical Research, The Australian National University, Canberra ACT 0200, Australia.)

Table S1. Kinetic parameters V (s^{-1}), K (μM), S ($s^{-1}.mM^{-1}$) and $S_{C/O}$ for C_3 species.

Species	V_C	K_C	S_C	V_O	K_O	S_O	$S_{C/O}$
Limonium antonii	2.4	8.7	276	0.976	397	2.46	112
Limonium artruchium	3	9.4	319	0.909	321	2.83	113
Limonium balearicum	3.6	9.7	371	1.24	346	3.58	103
Limonium barceloi	3.4	9.3	366	1.19	346	3.44	106
Limonium biflorum	2.4	8	300	0.845	316	2.67	112
Limonium companyonis	3.3	8.9	371	1.42	429	3.31	112
Limonium echioides	3.9	10.7	364	1.46	427	3.42	106
Limonium ejulabilis	2	7.6	263	0.94	415	2.27	116
Limonium gibertii	2.5	9.1	275	1.06	431	2.46	111
Limonium grosii	2.9	8.1	358	1.04	328	3.17	113
Limonium gymnesicum	2.4	8.2	293	0.936	388	2.41	121
Limonium latebracteatum	2.7	8.8	307		344		
Limonium leonardi	2.8	8.8	318	1.27	438	2.90	110
Limonium magallufianum	2.6	7	371	0.997	297	3.36	109
Limonium retusum	2.1	7.1	296	0.968	396	2.44	121
Limonium stenophyllum	2.6	8.4	310	0.976	457	2.46	
Limonium virgatum	2.4	8.5	282	0.909	381	2.83	
Mean	2.76	8.61	320	1.09	380	2.92	112
Standard Error	0.13	0.23	9	0.05	12	0.13	1
Aegilops biuncialis	3.2	16.8	190	0.932	470	1.98	96.3
Aegilops comosa	2.86	13.5	212	0.722	360	2.01	106
Aegilops cylindrica	3.68	13.7	269	1.12	451	2.48	109
Aegilops juvenalis	3.25	20.6	158	0.86	492	1.75	90.4
Aegilops speltoides	3.24	16.5	196	0.86	447	1.92	102
Aegilops tauschii	2.86	14.9	192	0.892	495	1.80	107
Aegilops triuncialis	2.62	12.8	205	0.754	380	1.98	103
Aegilops uniaristata	2.7	13.8	196	0.865	450	1.92	102
Aegilops vavilovii	3.32	13.3	250	0.835	363	2.30	109
Mean	3.08	15.1	207	0.87	434	2.01	103
Standard Error	0.11	0.8	11	0.04	18	0.08	2
Oryza barthii	2.5	14	179	0.798	479	1.67	107
Oryza eichingeri	2.5	14.1	177	1.01	612	1.66	107
Oryza glaberrima	2.7	14.9	181	1.01	586	1.73	105
Oryza glumaepatula	2.4	15.2	158	0.693	484	1.45	109
Oryza longistaminata	2.2	15.1	146	1.02	757	1.35	108
Oryza meridionalis	2.6	14.6	178	0.636	382	1.66	107
Oryza nivara	2.7	15.6	173	0.991	611	1.62	107
Oryza punctata	2.7	14.9	181	0.571	342	1.87	97
Oryza sativa	2.4	8.9	265	0.941	369	2.65	100
Mean	2.52	14.1	182	0.85	514	1.74	105
Standard Error	0.06	0.7	11	0.06	46	0.12	1
Puccinellia distans	5.4	22.2	243	1.14	488	2.34	104
Puccinellia lemmonii	5.2	28.1	185	2.06	1120	1.81	102
Puccinellia maritima	5.4	20.8	260	1.65	676	2.45	106
Puccinellia nuttalliana	4	25.2	159	1.08	717	1.51	105
Mean	5.00	24.0	212	1.48	750	2.03	104
Standard Error	0.34	1.6	24	0.23	133	0.22	1
Agriophyllum squarrosum	2.8	15.4	182	0.656	339	1.94	93.9

Agrostis scabra	3.6	22	164	1.04	653	1.59	103
Agrostis stolonifera	5.2	25.3	206	1.57	802	1.96	105
Amphicarpaea bracteata	4	29	138	0.978	693	1.41	97.5
Arabidopsis thaliana	3.61	9.9	364		333		
Arctagrostis latifolia	5.8	21	276	1.31	497	2.63	105
Artemisia myriantha	3.1	26.4	117	0.903	844	1.07	110
Artemisia vulgaris L.	3.9	31.9	122	0.73	626	1.16	105
Atriplex glabriuscula		27			328		
Avena sativa	2.3	10.8	213			2.13	99.9
B. distachyon	2.05	11.9	172	0.613	396	1.55	111
Beta maritima							94.6
Beta vulgaris	2.9	13.9	209	0.916	401	2.10	99.4
Brassica oleracea	2.1	11.8	178			1.85	96.2
Bromus anomalus	2.9	16.9	172	0.833	494	1.70	101
Calamagrostis arundinacea	4.1	22.7	181	1.07	614	1.74	104
Calamagrostis canescens	2.5	15.2	164	0.992	594	1.67	98.5
Calamagrostis foliosa	3.5	20.9	167	0.527	330	1.59	105
Calamagrostis inexpansa	3.3	18.8	176	0.971	608	1.58	111
Calamagrostis nutkaensis	3.1	20.1	154	0.853	601	1.41	109
Capsicum annuum	1.9	9.6	198			2.06	96
Chenopodium alba	2.91	11.2	260	1.37	415	3.30	78.7
Chenopodium murale	4.4	23.8	185	0.6	354	1.70	109
Chenopodium petiolare	4.4	25.6	172	1.03	589	1.74	98.5
Chenopodium rubrum	4.1	14.5	283	0.998	346	2.89	97.8
Citrullus ecirrhosus	3.1	18.9	164	0.882	544	1.64	99.9
Citrullus lanatus	2.5	19.4	129	0.616	510	1.20	107
Coffee arabica	2.1	11	191			1.93	98.7
Crithmum maritimum	3.4	8.7	391		183		
Cucurbita maxima	2.2	9	244			2.48	98.4
Dactylis glomerata	3.2	10.7	299		453		
Deschampsia danthonioides	4.5	22.3	202	1.09	580	1.87	108
Desmodium cinereum	3	12.8	234	0.97	403	2.40	97.5
Desmodium intortum	3.3	14.2	232	0.927	394	2.35	98.7
Desmodium psilocarpum	3.6	15.6	231	1.09	452	2.41	95.9
Diplotaxis ibicensis							95.6
Elymus farctus	3.3	19.5	169	0.476	300	1.60	106
Erythrina flabelliformis	3.6	18.4	196	1.36	665	2.03	96.4
Espeletia schultzii		23.3					
Eucalyptus moorei	3.2	10	320		285		
Eucalyptus neglecta	2.5	7.9	316		230		
Euphorbia helioscopia	1.9	11.5	165	0.77	453	1.71	96.8
Euphorbia microsphaera	4.5	25.8	174	0.954	546	1.75	99.7
Festuca gigantea	5.1	31.2	163	0.902	595	1.51	108
Festuca pratensis	5.1	23.1	221	1.43	686	2.08	106
Flueggea suffruticosa	3.4	19.2	177	0.96	547	1.75	101
Foeniculum vulgare	4.4	20.7	213	1.14	512	2.25	94.3
Glycine canescens	2.6	17.2	151	0.914	587	1.56	97.1
Glycine max	2	10.3	195	1.25	475	2.10	92.9
H. vulgare	3.99	15.2	263	1.2	465	2.57	102
Helianthus annuus							73.6
Helianthus maximus		10					77
Hordeum brachyantherum	2.9	16.2	179	0.656	371	1.77	101
Hordeum murinum	4.2	21.5	195	0.993	511	1.95	100
Hordeum vulgare	2.4	9	267			2.93	91.0
Hypericum balearicum							93.6
Ipomoea batatas	2.5	12	208			2.12	98.5

Iris douglasiana	3.5	9.7	361		413		
Kundmannia sicula							89.2
Lablab purpureus	5.3	21.7	244	1.49	556	2.68	91.1
Lactua sativa	2.2	11.1	198			2.11	94
Lepidium campestre	3.4	15.8	215	0.778	336	2.32	92.8
Lolium multiflorum	4.5	29.1	155	1.14	740	1.55	99.9
Lolium perenne		16			500		80
Lolium rigidum	4.7	25	188	0.973	520	1.88	100
Lycopersicon ensculentum		8.2					82
Lysimachia minoricensis							93.8
Macrotyloma uniflorum	4.4	25.2	175	0.897	519	1.73	101
Manihot esculenta	1.9	10.6	180			1.75	103
Medicago sativa	1.7	12.7	134			1.55	86.3
Mentha aquatica							97.2
Mercurialis annua	3.4	17	200	0.87	417	2.09	95.7
Musa velutina	3.2	19	168	0.852	564	1.52	111
Nicotiana glauca Grah.							73.7
Nicotiana tabacum	3.37	9.96	338	1.19	291	3.91	86.5
Pallenis maritima	2.7	6.4	422		321		
Petroselinum cripsum		11.6					77
Phaseolus carteri	3.2	14.2	225	1.13	422	2.67	84.5
Phaseolus coccineus	3.9	15.6	250	1.18	491	2.40	104
Phaseolus lunatus	3.2	17.3	185	0.981	537	1.83	101
Phaseolus vulgaris	2.6	14	186	0.761	463	1.81	102
Pistacia lentiscus							97.2
Pisum sativum							90.2
Plantago lanceolata L.							77.3
Poa palustris	4.2	19.2	219	1.1	562	1.95	112
Pueraria montana	2.7	20.9	129	0.871	679	1.28	101
Rhamnus alaternus							94.7
Rhamnus ludovici-salvatoris							94.4
S. cereale	3.23	20.2	160	0.826	472	1.75	91.5
Sideritis cretica subsp. spicata	2	7.8	256		328		
Solanum lycopersicum	2.3	9.7	237			2.57	92.4
Solanum tuberosum	2	9.6	208			2.18	95.4
Sphenostylis stenocarpa	2.8	17.4	161	0.981	574	1.72	93.6
Spinacia oleracea	2.76	12.1	228	1.39	461	2.53	90.5
Steinchisma laxa	2.3	7.7	299	1.35	419	3.27	91.4
T. aestivum	3.45	16.8	205	0.917	429	2.15	95.7
T. dicoccon1	3.51	16.1	218	1.02	434	2.33	93.5
T. monococcum	3.18	14	227	0.877	401	2.18	104
T. timonovum	3.48	16.8	207	1.02	495	2.05	101
T. timopheevii	3.43	16.2	212	0.893	429	2.08	102
Tephrosia candida	2.2	15.9	138	0.667	481	1.41	97.8
Tephrosia purpurea	2.2	12.4	177	0.575	333	1.72	103
Tephrosia rhodesica	2.2	13.7	161	0.988	565	1.75	91.6
Tetragonium expansa		13			600		81
Teucrium heterophyllum	2.7	6.7	403		359		
Trachycarpus fortunei	2.8	9	311		364		
Trifolium repens		13.1			565		
Triticale	3.48	15.7	222	0.905	396	2.28	97.2
Triticum aestivum	3.18	13.1	243	1.39	543	2.55	95.4
Triticum baeoticum	3.8	19.7	193	1.8	905	2.01	96
Urtica atrovirens							90.2
Urtica membranacea							102

Table S2. Kinetic parameters V (s^{-1}), K (μM), S ($s^{-1}.mM^{-1}$) and $S_{C/O}$ for C_3, transitional and C_4 species.

**Species**		V_C	K_C	S_C	V_O	K_O	S_O	$S_{C/O}$
Flaveria cronquistii	C_3	3.04	10.3	297	2.34	431	3.51	84.6
Flaveria pringlei	C_3	2.80	12.2	229	1.61	321	2.63	86.9
Flaveria angustifolia	C_3-C_4	2.82	12.8	220			2.53	86.8
Flaveria anomala	C_3-C_4	3.8	10.7	355	2.75	605	4.56	77.9
Flaveria chloraefolia	C_3-C_4	3.35	12.4	270	2.46	740	3.31	81.6
Flaveria floridana	C_3-C_4	3.22	13.2	244	1.55	530	2.92	83.6
Flaveria linearis	C_3-C_4	3.43	12.5	274	1.46	415	3.51	78.1
Flaveria ramosissima	C_3-C_4	2.77	12	231	2.09	722	2.89	79.8
Flaveria sonorensis	C_3-C_4	2.69	10.2	264	2.46	785	3.13	84.3
Flaveria brownii	C_4 like	2.58	12.8	202	0.907	378	2.41	83.8
Flaveria palmeri	C_4 like	3.54	13.5	262	0.603	193	3.13	83.8
Flaveria vaginata	C_4 like	3.78	21.4	177	1.97	880	2.24	78.7
Flaveria australasica	C_4	3.84	22	175	0.697	309	2.62	77.2
Flaveria bidentis	C_4	4.13	20.0	206	1.48	530	2.61	78.8
Flaveria kochiana	C_4	3.68	22.7	162		150	2.11	77
Flaveria trinervia	C_4	3.85	18.2	212	2.15	671	2.73	77.7
Mean		3.33	14.8	236	1.72	521	2.91	81.3
Standard Error		0.12	1.1	13	0.17	58	0.16	0.9
Panicum bisulcatum	C_3	2.6	7.8	333	1.57	416	3.80	87.7
Panicum milioides	C_3-C_4	2.2	7.4	297	1.24	387	3.22	92.3
Panicum amarum	C_4	3.2	33.1	97	0.86	800	1.08	89.5
Panicum antidotale	C_4	3.9						74.5
Panicum coloratum	C_4	3.4	11.1	306	1.59	445	3.61	84.8
Panicum deustum	C_4	5	15.4	325	1.17	306	3.83	84.8
Panicum dichotomiflorum	C_4	3.1	36.3	85	1.41	154	0.92	92.6
Panicum milliaceum	C_4	2.1	7.2	292	1.13	313	3.65	79.9
Panicum monticola	C_4	5.3	18.2	291	1.97	543	3.67	79.4
Panicum phragmitoides	C_4	2.8	25.1	112	0.707	687	1.04	107
Panicum virgatum	C_4	3.3	12.7	260	0.854	271	3.15	82.6
Mean		3.35	17.4	240	1.25	571	2.80	86.6
Standard Error		0.31	3.38	32	0.12	120	0.40	2.9
Amaranthus edulis	C_4	4.14	18.2	227	0.847	289	2.94	77.5
Amaranthus hybridus	C_4	3.8	16	238	1.85	640	2.97	80
C. dactylon	C_4		21			402		89.2
Cenchrus ciliaris	C_4	6	19	316	2.1	470	4.52	69.9
Chrysanthellum indicum	C_4	4.7	28.1	167	1.21	598	2.03	82.4
Echinochloa crus-galli	C_4		18.4					83
Eragrostis tef	C_4	7.1	34.9	203	1.46	640	2.29	89
Megathyrsus maximus	C_4	5.3	13.9	381	1.25	265	4.75	80.3
P. dilatatum	C_4		19.9			415		88
Potulaca oleraca	C_4	5.9	13.6	434			5.56	78
Saccharum officinarum	C_4	3.9	26.3	148			1.80	82.2
Setaria italica	C_4		32.1					58
Setaria viridis	C_4	5.67	18.1	313	2.77	619	4.31	72.7
Sorghum bicolor	C_4	5.4	29.9	181			2.58	70
Urochloa mosambicensis	C_4	5.7	14.8	385	2.14	464	4.67	82.5
Urochloa panicoides	C_4	5.6	15.4	364	2.04	444	4.64	78.3
Z. japonica	C_4		18.5			403		84.1
Zea mays	C_4	4.19	30.6	137	0.920	596	1.68	81.6
Mean		5.18	21.6	269	1.66	480	3.44	79.0
Standard Error		0.27	1.6	28	0.20	36	0.37	1.8

Appendix

Derivation of the kinetic equations: The concentrations used in the kinetic equations are E: activated form of the enzyme; R: unbound RuBP; ER: RuBisCO…RuBP complex; ER^*: RuBisCO…enediolate of RuBP complex; C: free CO_2; O: free O_2; ERC: RuBisCO…carboxylated intermediate complex; ERO: RuBisCO…oxygenated intermediate complex; ERP: RuBisCO…carboxylated product complex; ERX: RuBisCO…oxygenated product complex; G: 3-phosphoglyceric acid; Q: 2-phospho-glycolate.

The mass balance equation for the kinetic mechanism (Fig. 1) is given by (E_t is the total activated enzyme concentration)

$$E + ER + ER^* + ERC + ERO + EP + EX - E_t = 0 \qquad \text{(A1)}$$

The steady state ordinary differential equations (ODEs) for this kinetic scheme are

$$d\,ER/dt = k_1\,E \cdot R - (k_2 + k_3)ER + k_4 ER^* = 0 \qquad \text{(A2)}$$

$$d\,ER^*/dt = k_3\,ER - (k_4 + k_5 C + k_{11} O)ER^* + k_6 ERC + k_{12} ERO = 0 \qquad \text{(A3)}$$

$$d\,ERC/dt = k_5\,C \cdot ER^* - (k_6 + k_7)ERC + k_8 EP = 0 \qquad \text{(A4)}$$

$$d\,EP/dt = k_7\,ERC - (k_8 + k_9)EP + k_{10} E \cdot G \cdot G = 0 \qquad \text{(A5)}$$

$$d\,ERO/dt = k_{11}\,O \cdot ER^* - (k_{12} + k_{13})ERO + k_{14} EX = 0 \qquad \text{(A6)}$$

$$d\,EX/dt = k_{13}\,ERO - (k_{14} + k_{15})EX + k_{16} E \cdot G \cdot Q = 0 \qquad \text{(A7)}$$

It is convenient to define the following constants:

$$\alpha_C = (k_9 + k_8)/k_7 \qquad \alpha_O = (k_{15} + k_{14})/k_{13}$$
$$\beta_C = k_9 + k_6 \alpha_C \qquad \beta_O = k_{15} + k_{12} \alpha_O$$

From the above steady state ODEs we can readily express the concentrations of free enzyme E and all reaction intermediates in terms of the product complexes (either EP or EX). For the carboxylation reaction (EP) we obtain (assuming only that product release is "irreversible" i.e. $k_{10} = k_{16} = 0$):

Summing (A2) to (A7): $$E = \frac{k_2 ER + k_9 EP + k_{15} EX}{k_1 R} \qquad \text{(A8)}$$

From (A5): $ERC = \alpha_C EP$ (A9)

From (A7): $ERO = \alpha_O EX$ (A10)

From (A4) + (A5) and (A9): $$ER^* = \frac{k_9 EP + k_6 ERC}{k_5 C} = \frac{\beta_C}{k_5 C} EP \qquad \text{(A11)}$$

From (A6) + (A7), (A10) and (A11): $$EX = \frac{k_{11} O \cdot ER^*}{\beta_O} = \frac{\beta_C k_{11} O}{\beta_O k_5 C} EP \qquad \text{(A12)}$$

From (A10) and (A12): $$ERO = \alpha_O EX = \frac{\alpha_O \beta_C k_{11} O}{\beta_O k_5 C} EP \qquad \text{(A13)}$$

From (A2), (A8), (A11) and (A12): $ER = \dfrac{k_9 EP + k_{15} EX + k_4 ER^*}{k_3}$

$$= \frac{k_9}{k_3} EP + \frac{\beta_C k_{11} k_{15} O}{\beta_O k_3 k_5 C} EP + \frac{k_4 \beta_C}{k_3 k_5 C} EP \qquad \text{(A14)}$$

Substituting (A8)-(A14) into (A1) and factorizing we get the steady state equation in the form

$$\frac{E_t}{EP} = 1 + f_1^C + \frac{f_2^C}{R} + \frac{f_3^C}{C} + \frac{f_4^C}{RC} \qquad \text{(A15)}$$

where the coefficients f_i^C are given by

$$f_1^C = \frac{k_9 + k_3 \alpha_C}{k_3} \qquad \text{(A16a)}$$

$$f_2^C = \frac{k_9 (k_2 + k_3)}{k_1 k_3} \qquad \text{(A16b)}$$

$$f_3^C = \frac{\beta_C}{k_3 k_5} \left(k_3 + k_4 + \frac{(k_3 + k_{15} + k_3 \alpha_O) k_{11} O}{\beta_O} \right) \qquad \text{(A16c)}$$

$$f_4^C = \frac{\beta_C}{k_1 k_3 k_5} \left(k_2 k_4 + \frac{(k_2 + k_3) k_{11} k_{15} O}{\beta_O} \right) \qquad \text{(A16d)}$$

The rate of CO_2 consumption is given by

$$V_C = -\frac{dC}{dt} = k_5 C \cdot ER^* - k_6 ERC = (k_9 + k_6 \alpha_C) EP - k_6 \alpha_C EP = k_9 EP \qquad \text{(A17)}$$

Rewriting (A15) in terms of EP and substituting the result into (A17) gives

$$V_C = \frac{k_9 E_t RC}{f_4^C + f_3^C R + f_2^C C + (f_1^C + 1) RC} \qquad \text{(A18)}$$

When both substrates, *R* and *C*, are saturating the maximum rate of CO_2 consumption, $V_{\max}^C$, is obtained as,

$$V_{\max}^C = \frac{k_9}{1 + f_1^C} E_t = k_{cat}^C E_t \qquad \text{(A19)}$$

Substituting (A16a) into (A19) and rearranging we get for k_{cat}^C in terms of the rate constants k_i

$$k_{cat}^C = \frac{k_3 k_7 k_9}{k_3 k_7 + k_3 k_8 + k_3 k_9 + k_7 k_9}$$

Finally, rewriting (A18) in terms of $V_{\max}^C$ gives the familiar (e.g. (Farquhar, 1979)) general form of the steady state rate equation,

$$V_C = \frac{V_{\max}^C R \cdot C}{K_3^C + K_2^C R + K_1^C C + R \cdot C} \qquad \text{(A20)}$$

where $K_i^C = f_{i+1}^C / (1 + f_1^C)$. (A21)

It immediately follows that the rate of oxygen consumption by the enzyme can be written as

$$V_O = \frac{V_{\max}^O R \cdot O}{K_3^O + K_2^O R + K_1^O O + R \cdot O}$$

where $V_{\max}^O = k_{cat}^O E_t$ and $k_{cat}^O = \frac{k_{15}}{1 + f_1^O} = \frac{k_3 k_{13} k_{15}}{k_3 k_{13} + k_3 k_{14} + k_3 k_{15} + k_{13} k_{15}}$.

The Michaelis-Menten equation (A20) for the single substrate *C* when *R* is saturating becomes

$$V_C = \frac{V_{\max}^C C}{K_2^C + C}$$

From (A21), (A16a) and (A16c):

$$K_2^C = \frac{f_3^C}{1 + f_1^C} = \frac{\beta_C}{(1 + f_1^C) k_3 k_5} \left[k_3 + k_4 + \frac{(k_3 + k_{15} + k_3 \alpha_O) k_{11} O}{\beta_O} \right] \qquad \text{(A22)}$$

Substituting $f_1^O = \frac{k_{15} + k_3 \alpha_O}{k_3}$, $K_R = \frac{k_3}{k_3 + k_4}$, $\gamma_C = \frac{\alpha_C}{1 + f_1^C}$ and $\gamma_O = \frac{\alpha_O}{1 + f_1^O}$
into (A22) yields the Michaelis constant in the presence of the other (*O*) substrate

$$K_2^C = K_C \left(1 + \frac{O}{K_O} \right)$$

where $K_C = \frac{k_9 + k_6 \alpha_C}{(1 + f_1^C) K_R k_5} = \frac{k_{cat}^C + \gamma_C k_6}{K_R k_5}$ (A23)

and $K_O = \frac{k_{15} + k_{12} \alpha_O}{(1 + f_1^O) K_R k_{11}} = \frac{k_{cat}^O + \gamma_O k_{12}}{K_R k_{11}}$ (A24)

The specificities of each of the reactions are then

$$S_C = \frac{k_{cat}^C}{K_C} = \frac{k_{cat}^C K_R k_5}{k_{cat}^C + \gamma_C k_6} \qquad \text{and} \qquad S_O = \frac{k_{cat}^O}{K_O} = \frac{k_{cat}^O K_R k_{11}}{k_{cat}^O + \gamma_O k_{12}}$$

and so the specificity of carboxylation relative to oxygenation (relative specificity) is given by

$$S_{C/O} = \frac{S_C}{S_O} = \frac{k_5 k_{cat}^C (k_{cat}^O + \gamma_O k_{12})}{k_{11} k_{cat}^O (k_{cat}^C + \gamma_C k_6)} \tag{A25}$$

In terms of the rate constants we find the coefficients of k_6 and k_{12}:

$$\gamma_C = \frac{\alpha_C}{1 + f_1^C} = \frac{(k_8 + k_9) k_{cat}^C}{k_7 k_9} = \frac{k_3 k_8 + k_3 k_9}{k_3 k_7 + k_3 k_8 + k_3 k_9 + k_7 k_9} \tag{A26}$$

$$\gamma_O = \frac{\alpha_O}{1 + f_1^O} = \frac{(k_{14} + k_{15}) k_{cat}^O}{k_{13} k_{15}} = \frac{k_3 k_{14} + k_3 k_{15}}{k_3 k_{13} + k_3 k_{14} + k_3 k_{15} + k_{13} k_{15}} \tag{A27}$$

Thus the range of γ is limited to [0,1].
