# Supplementary material for: The Coevolution of RuBisCO, Photorespiration, and Carbon Concentrating Mechanisms in Higher Plants
Source: Front Plant Sci. 2021 Sep 1;12:662425. doi: 10.3389/fpls.2021.662425 (PMC8440988; doi:10.3389/fpls.2021.662425)
Supplement: Supplementary file 2 [file Table_1.DOCX]

**Supplementary Material for:** **The Co-evolution of RuBisCO, Photorespiration and Carbon Concentrating Mechanisms in Higher Plants** (**Peter L. Cummins,** Department of Genome Sciences, John Curtin School of Medical Research, The Australian National University, Canberra ACT 0200, Australia.)

**Table S1.** Kinetic parameters *V* (s^-1^), *K* (μM), *S* (s^-1^.mM^-1^) and *S*_C/O_ for C_3_ species.

| **Species** | ***V*_C_** | ***K*_C_** | ***S*_C_** | ***V*_O_** | ***K*_O_** | ***S*_O_** | ***S*_C/O_** |
| --- | --- | --- | --- | --- | --- | --- | --- |
| *Limonium antonii* | 2.4 | 8.7 | 276 | 0.976 | 397 | 2.46 | 112 |
| *Limonium artruchium* | 3 | 9.4 | 319 | 0.909 | 321 | 2.83 | 113 |
| *Limonium balearicum* | 3.6 | 9.7 | 371 | 1.24 | 346 | 3.58 | 103 |
| *Limonium barceloi* | 3.4 | 9.3 | 366 | 1.19 | 346 | 3.44 | 106 |
| *Limonium biflorum* | 2.4 | 8 | 300 | 0.845 | 316 | 2.67 | 112 |
| *Limonium companyonis* | 3.3 | 8.9 | 371 | 1.42 | 429 | 3.31 | 112 |
| *Limonium echioides* | 3.9 | 10.7 | 364 | 1.46 | 427 | 3.42 | 106 |
| *Limonium ejulabilis* | 2 | 7.6 | 263 | 0.94 | 415 | 2.27 | 116 |
| *Limonium gibertii* | 2.5 | 9.1 | 275 | 1.06 | 431 | 2.46 | 111 |
| *Limonium grosii* | 2.9 | 8.1 | 358 | 1.04 | 328 | 3.17 | 113 |
| *Limonium gymnesicum* | 2.4 | 8.2 | 293 | 0.936 | 388 | 2.41 | 121 |
| *Limonium latebracteatum* | 2.7 | 8.8 | 307 |  | 344 |  |  |
| *Limonium leonardi* | 2.8 | 8.8 | 318 | 1.27 | 438 | 2.90 | 110 |
| *Limonium magallufianum* | 2.6 | 7 | 371 | 0.997 | 297 | 3.36 | 109 |
| *Limonium retusum* | 2.1 | 7.1 | 296 | 0.968 | 396 | 2.44 | 121 |
| *Limonium stenophyllum* | 2.6 | 8.4 | 310 | 0.976 | 457 | 2.46 |  |
| *Limonium virgatum* | 2.4 | 8.5 | 282 | 0.909 | 381 | 2.83 |  |
| Mean | 2.76 | 8.61 | 320 | 1.09 | 380 | 2.92 | 112 |
| Standard Error | 0.13 | 0.23 | 9 | 0.05 | 12 | 0.13 | 1 |
| *Aegilops biuncialis* | 3.2 | 16.8 | 190 | 0.932 | 470 | 1.98 | 96.3 |
| *Aegilops comosa* | 2.86 | 13.5 | 212 | 0.722 | 360 | 2.01 | 106 |
| *Aegilops cylindrica* | 3.68 | 13.7 | 269 | 1.12 | 451 | 2.48 | 109 |
| *Aegilops juvenalis* | 3.25 | 20.6 | 158 | 0.86 | 492 | 1.75 | 90.4 |
| *Aegilops speltoides* | 3.24 | 16.5 | 196 | 0.86 | 447 | 1.92 | 102 |
| *Aegilops tauschii* | 2.86 | 14.9 | 192 | 0.892 | 495 | 1.80 | 107 |
| *Aegilops triuncialis* | 2.62 | 12.8 | 205 | 0.754 | 380 | 1.98 | 103 |
| *Aegilops uniaristata* | 2.7 | 13.8 | 196 | 0.865 | 450 | 1.92 | 102 |
| *Aegilops vavilovii* | 3.32 | 13.3 | 250 | 0.835 | 363 | 2.30 | 109 |
| Mean | 3.08 | 15.1 | 207 | 0.87 | 434 | 2.01 | 103 |
| Standard Error | 0.11 | 0.8 | 11 | 0.04 | 18 | 0.08 | 2 |
| *Oryza barthii* | 2.5 | 14 | 179 | 0.798 | 479 | 1.67 | 107 |
| *Oryza eichingeri* | 2.5 | 14.1 | 177 | 1.01 | 612 | 1.66 | 107 |
| *Oryza glaberrima* | 2.7 | 14.9 | 181 | 1.01 | 586 | 1.73 | 105 |
| *Oryza glumaepatula* | 2.4 | 15.2 | 158 | 0.693 | 484 | 1.45 | 109 |
| *Oryza longistaminata* | 2.2 | 15.1 | 146 | 1.02 | 757 | 1.35 | 108 |
| *Oryza meridionalis* | 2.6 | 14.6 | 178 | 0.636 | 382 | 1.66 | 107 |
| *Oryza nivara* | 2.7 | 15.6 | 173 | 0.991 | 611 | 1.62 | 107 |
| *Oryza punctata* | 2.7 | 14.9 | 181 | 0.571 | 342 | 1.87 | 97 |
| *Oryza sativa* | 2.4 | 8.9 | 265 | 0.941 | 369 | 2.65 | 100 |
| Mean | 2.52 | 14.1 | 182 | 0.85 | 514 | 1.74 | 105 |
| Standard Error | 0.06 | 0.7 | 11 | 0.06 | 46 | 0.12 | 1 |
| *Puccinellia distans* | 5.4 | 22.2 | 243 | 1.14 | 488 | 2.34 | 104 |
| *Puccinellia lemmonii* | 5.2 | 28.1 | 185 | 2.06 | 1120 | 1.81 | 102 |
| *Puccinellia maritima* | 5.4 | 20.8 | 260 | 1.65 | 676 | 2.45 | 106 |
| *Puccinellia nuttalliana* | 4 | 25.2 | 159 | 1.08 | 717 | 1.51 | 105 |
| Mean | 5.00 | 24.0 | 212 | 1.48 | 750 | 2.03 | 104 |
| Standard Error | 0.34 | 1.6 | 24 | 0.23 | 133 | 0.22 | 1 |
| *Agriophyllum squarrosum* | 2.8 | 15.4 | 182 | 0.656 | 339 | 1.94 | 93.9 |
| *Agrostis scabra* | 3.6 | 22 | 164 | 1.04 | 653 | 1.59 | 103 |
| *Agrostis stolonifera* | 5.2 | 25.3 | 206 | 1.57 | 802 | 1.96 | 105 |
| *Amphicarpaea bracteata* | 4 | 29 | 138 | 0.978 | 693 | 1.41 | 97.5 |
| *Arabidopsis thaliana* | 3.61 | 9.9 | 364 |  | 333 |  |  |
| *Arctagrostis latifolia* | 5.8 | 21 | 276 | 1.31 | 497 | 2.63 | 105 |
| *Artemisia myriantha* | 3.1 | 26.4 | 117 | 0.903 | 844 | 1.07 | 110 |
| *Artemisia vulgaris L.* | 3.9 | 31.9 | 122 | 0.73 | 626 | 1.16 | 105 |
| *Atriplex glabriuscula* |  | 27 |  |  | 328 |  |  |
| *Avena sativa* | 2.3 | 10.8 | 213 |  |  | 2.13 | 99.9 |
| *B. distachyon* | 2.05 | 11.9 | 172 | 0.613 | 396 | 1.55 | 111 |
| *Beta maritima* |  |  |  |  |  |  | 94.6 |
| *Beta vulgaris* | 2.9 | 13.9 | 209 | 0.916 | 401 | 2.10 | 99.4 |
| *Brassica oleracea* | 2.1 | 11.8 | 178 |  |  | 1.85 | 96.2 |
| *Bromus anomalus* | 2.9 | 16.9 | 172 | 0.833 | 494 | 1.70 | 101 |
| *Calamagrostis arundinacea* | 4.1 | 22.7 | 181 | 1.07 | 614 | 1.74 | 104 |
| *Calamagrostis canescens* | 2.5 | 15.2 | 164 | 0.992 | 594 | 1.67 | 98.5 |
| *Calamagrostis foliosa* | 3.5 | 20.9 | 167 | 0.527 | 330 | 1.59 | 105 |
| *Calamagrostis inexpansa* | 3.3 | 18.8 | 176 | 0.971 | 608 | 1.58 | 111 |
| *Calamagrostis nutkaensis* | 3.1 | 20.1 | 154 | 0.853 | 601 | 1.41 | 109 |
| *Capsicum annuum* | 1.9 | 9.6 | 198 |  |  | 2.06 | 96 |
| *Chenopodium alba* | 2.91 | 11.2 | 260 | 1.37 | 415 | 3.30 | 78.7 |
| *Chenopodium murale* | 4.4 | 23.8 | 185 | 0.6 | 354 | 1.70 | 109 |
| *Chenopodium petiolare* | 4.4 | 25.6 | 172 | 1.03 | 589 | 1.74 | 98.5 |
| *Chenopodium rubrum* | 4.1 | 14.5 | 283 | 0.998 | 346 | 2.89 | 97.8 |
| *Citrullus ecirrhosus* | 3.1 | 18.9 | 164 | 0.882 | 544 | 1.64 | 99.9 |
| *Citrullus lanatus* | 2.5 | 19.4 | 129 | 0.616 | 510 | 1.20 | 107 |
| *Coffee arabica* | 2.1 | 11 | 191 |  |  | 1.93 | 98.7 |
| *Crithmum maritimum* | 3.4 | 8.7 | 391 |  | 183 |  |  |
| *Cucurbita maxima* | 2.2 | 9 | 244 |  |  | 2.48 | 98.4 |
| *Dactylis glomerata* | 3.2 | 10.7 | 299 |  | 453 |  |  |
| *Deschampsia danthonioides* | 4.5 | 22.3 | 202 | 1.09 | 580 | 1.87 | 108 |
| *Desmodium cinereum* | 3 | 12.8 | 234 | 0.97 | 403 | 2.40 | 97.5 |
| *Desmodium intortum* | 3.3 | 14.2 | 232 | 0.927 | 394 | 2.35 | 98.7 |
| *Desmodium psilocarpum* | 3.6 | 15.6 | 231 | 1.09 | 452 | 2.41 | 95.9 |
| *Diplotaxis ibicensis* |  |  |  |  |  |  | 95.6 |
| *Elymus farctus* | 3.3 | 19.5 | 169 | 0.476 | 300 | 1.60 | 106 |
| *Erythrina flabelliformis* | 3.6 | 18.4 | 196 | 1.36 | 665 | 2.03 | 96.4 |
| *Espeletia schultzii* |  | 23.3 |  |  |  |  |  |
| *Eucalyptus moorei* | 3.2 | 10 | 320 |  | 285 |  |  |
| *Eucalyptus neglecta* | 2.5 | 7.9 | 316 |  | 230 |  |  |
| *Euphorbia helioscopia* | 1.9 | 11.5 | 165 | 0.77 | 453 | 1.71 | 96.8 |
| *Euphorbia microsphaera* | 4.5 | 25.8 | 174 | 0.954 | 546 | 1.75 | 99.7 |
| *Festuca gigantea* | 5.1 | 31.2 | 163 | 0.902 | 595 | 1.51 | 108 |
| *Festuca pratensis* | 5.1 | 23.1 | 221 | 1.43 | 686 | 2.08 | 106 |
| *Flueggea suffruticosa* | 3.4 | 19.2 | 177 | 0.96 | 547 | 1.75 | 101 |
| *Foeniculum vulgare* | 4.4 | 20.7 | 213 | 1.14 | 512 | 2.25 | 94.3 |
| *Glycine canescens* | 2.6 | 17.2 | 151 | 0.914 | 587 | 1.56 | 97.1 |
| *Glycine max* | 2 | 10.3 | 195 | 1.25 | 475 | 2.10 | 92.9 |
| *H. vulgare* | 3.99 | 15.2 | 263 | 1.2 | 465 | 2.57 | 102 |
| *Helianthus annuus* |  |  |  |  |  |  | 73.6 |
| *Helianthus maximus* |  | 10 |  |  |  |  | 77 |
| *Hordeum brachyantherum* | 2.9 | 16.2 | 179 | 0.656 | 371 | 1.77 | 101 |
| *Hordeum murinum* | 4.2 | 21.5 | 195 | 0.993 | 511 | 1.95 | 100 |
| *Hordeum vulgare* | 2.4 | 9 | 267 |  |  | 2.93 | 91.0 |
| *Hypericum balearicum* |  |  |  |  |  |  | 93.6 |
| *Ipomoea batatas* | 2.5 | 12 | 208 |  |  | 2.12 | 98.5 |
| *Iris douglasiana* | 3.5 | 9.7 | 361 |  | 413 |  |  |
| *Kundmannia sicula* |  |  |  |  |  |  | 89.2 |
| *Lablab purpureus* | 5.3 | 21.7 | 244 | 1.49 | 556 | 2.68 | 91.1 |
| *Lactua sativa* | 2.2 | 11.1 | 198 |  |  | 2.11 | 94 |
| *Lepidium campestre* | 3.4 | 15.8 | 215 | 0.778 | 336 | 2.32 | 92.8 |
| *Lolium multiflorum* | 4.5 | 29.1 | 155 | 1.14 | 740 | 1.55 | 99.9 |
| *Lolium perenne* |  | 16 |  |  | 500 |  | 80 |
| *Lolium rigidum* | 4.7 | 25 | 188 | 0.973 | 520 | 1.88 | 100 |
| *Lycopersicon ensculentum* |  | 8.2 |  |  |  |  | 82 |
| *Lysimachia minoricensis* |  |  |  |  |  |  | 93.8 |
| *Macrotyloma uniflorum* | 4.4 | 25.2 | 175 | 0.897 | 519 | 1.73 | 101 |
| *Manihot esculenta* | 1.9 | 10.6 | 180 |  |  | 1.75 | 103 |
| *Medicago sativa* | 1.7 | 12.7 | 134 |  |  | 1.55 | 86.3 |
| *Mentha aquatica* |  |  |  |  |  |  | 97.2 |
| *Mercurialis annua* | 3.4 | 17 | 200 | 0.87 | 417 | 2.09 | 95.7 |
| *Musa velutina* | 3.2 | 19 | 168 | 0.852 | 564 | 1.52 | 111 |
| *Nicotiana glauca Grah.* |  |  |  |  |  |  | 73.7 |
| *Nicotiana tabacum* | 3.37 | 9.96 | 338 | 1.19 | 291 | 3.91 | 86.5 |
| *Pallenis maritima* | 2.7 | 6.4 | 422 |  | 321 |  |  |
| *Petroselinum cripsum* |  | 11.6 |  |  |  |  | 77 |
| *Phaseolus carteri* | 3.2 | 14.2 | 225 | 1.13 | 422 | 2.67 | 84.5 |
| *Phaseolus coccineus* | 3.9 | 15.6 | 250 | 1.18 | 491 | 2.40 | 104 |
| *Phaseolus lunatus* | 3.2 | 17.3 | 185 | 0.981 | 537 | 1.83 | 101 |
| *Phaseolus vulgaris* | 2.6 | 14 | 186 | 0.761 | 463 | 1.81 | 102 |
| *Pistacia lentiscus* |  |  |  |  |  |  | 97.2 |
| *Pisum sativum* |  |  |  |  |  |  | 90.2 |
| *Plantago lanceolata L.* |  |  |  |  |  |  | 77.3 |
| *Poa palustris* | 4.2 | 19.2 | 219 | 1.1 | 562 | 1.95 | 112 |
| *Pueraria montana* | 2.7 | 20.9 | 129 | 0.871 | 679 | 1.28 | 101 |
| *Rhamnus alaternus* |  |  |  |  |  |  | 94.7 |
| *Rhamnus ludovici-salvatoris* |  |  |  |  |  |  | 94.4 |
| *S. cereale* | 3.23 | 20.2 | 160 | 0.826 | 472 | 1.75 | 91.5 |
| *Sideritis cretica subsp. spicata* | 2 | 7.8 | 256 |  | 328 |  |  |
| *Solanum lycopersicum* | 2.3 | 9.7 | 237 |  |  | 2.57 | 92.4 |
| *Solanum tuberosum* | 2 | 9.6 | 208 |  |  | 2.18 | 95.4 |
| *Sphenostylis stenocarpa* | 2.8 | 17.4 | 161 | 0.981 | 574 | 1.72 | 93.6 |
| *Spinacia oleracea* | 2.76 | 12.1 | 228 | 1.39 | 461 | 2.53 | 90.5 |
| *Steinchisma laxa* | 2.3 | 7.7 | 299 | 1.35 | 419 | 3.27 | 91.4 |
| *T. aestivum* | 3.45 | 16.8 | 205 | 0.917 | 429 | 2.15 | 95.7 |
| *T. dicoccon1* | 3.51 | 16.1 | 218 | 1.02 | 434 | 2.33 | 93.5 |
| *T. monococcum* | 3.18 | 14 | 227 | 0.877 | 401 | 2.18 | 104 |
| *T. timonovum* | 3.48 | 16.8 | 207 | 1.02 | 495 | 2.05 | 101 |
| *T. timopheevii* | 3.43 | 16.2 | 212 | 0.893 | 429 | 2.08 | 102 |
| *Tephrosia candida* | 2.2 | 15.9 | 138 | 0.667 | 481 | 1.41 | 97.8 |
| *Tephrosia purpurea* | 2.2 | 12.4 | 177 | 0.575 | 333 | 1.72 | 103 |
| *Tephrosia rhodesica* | 2.2 | 13.7 | 161 | 0.988 | 565 | 1.75 | 91.6 |
| *Tetragonium expansa* |  | 13 |  |  | 600 |  | 81 |
| *Teucrium heterophyllum* | 2.7 | 6.7 | 403 |  | 359 |  |  |
| *Trachycarpus fortunei* | 2.8 | 9 | 311 |  | 364 |  |  |
| *Trifolium repens* |  | 13.1 |  |  | 565 |  |  |
| *Triticale* | 3.48 | 15.7 | 222 | 0.905 | 396 | 2.28 | 97.2 |
| *Triticum aestivum* | 3.18 | 13.1 | 243 | 1.39 | 543 | 2.55 | 95.4 |
| *Triticum baeoticum* | 3.8 | 19.7 | 193 | 1.8 | 905 | 2.01 | 96 |
| *Urtica atrovirens* |  |  |  |  |  |  | 90.2 |
| *Urtica membranacea* |  |  |  |  |  |  | 102 |

**Table S2.** Kinetic parameters *V* (s^-1^), *K* (μM), *S* (s^-1^.mM^-1^) and *S*_C/O_ for C_3_, transitional and C_4_ species.

| **Species** |  | ***V*_C_** | ***K*_C_** | ***S*_C_** | ***V*_O_** | ***K*_O_** | ***S*_O_** | ***S*_C/O_** |
| --- | --- | --- | --- | --- | --- | --- | --- | --- |
| *Flaveria cronquistii* | C_3_ | 3.04 | 10.3 | 297 | 2.34 | 431 | 3.51 | 84.6 |
| *Flaveria pringlei* | C_3_ | 2.80 | 12.2 | 229 | 1.61 | 321 | 2.63 | 86.9 |
| *Flaveria angustifolia* | C_3_-C_4_ | 2.82 | 12.8 | 220 |  |  | 2.53 | 86.8 |
| *Flaveria anomala* | C_3_-C_4_ | 3.8 | 10.7 | 355 | 2.75 | 605 | 4.56 | 77.9 |
| *Flaveria chloraefolia* | C_3_-C_4_ | 3.35 | 12.4 | 270 | 2.46 | 740 | 3.31 | 81.6 |
| *Flaveria floridana* | C_3_-C_4_ | 3.22 | 13.2 | 244 | 1.55 | 530 | 2.92 | 83.6 |
| *Flaveria linearis* | C_3_-C_4_ | 3.43 | 12.5 | 274 | 1.46 | 415 | 3.51 | 78.1 |
| *Flaveria ramosissima* | C_3_-C_4_ | 2.77 | 12 | 231 | 2.09 | 722 | 2.89 | 79.8 |
| *Flaveria sonorensis* | C_3_-C_4_ | 2.69 | 10.2 | 264 | 2.46 | 785 | 3.13 | 84.3 |
| *Flaveria brownii* | C_4_ like | 2.58 | 12.8 | 202 | 0.907 | 378 | 2.41 | 83.8 |
| *Flaveria palmeri* | C_4_ like | 3.54 | 13.5 | 262 | 0.603 | 193 | 3.13 | 83.8 |
| *Flaveria vaginata* | C_4_ like | 3.78 | 21.4 | 177 | 1.97 | 880 | 2.24 | 78.7 |
| *Flaveria australasica* | C_4_ | 3.84 | 22 | 175 | 0.697 | 309 | 2.62 | 77.2 |
| *Flaveria bidentis* | C_4_ | 4.13 | 20.0 | 206 | 1.48 | 530 | 2.61 | 78.8 |
| *Flaveria kochiana* | C_4_ | 3.68 | 22.7 | 162 |  | 150 | 2.11 | 77 |
| *Flaveria trinervia* | C_4_ | 3.85 | 18.2 | 212 | 2.15 | 671 | 2.73 | 77.7 |
| Mean |  | 3.33 | 14.8 | 236 | 1.72 | 521 | 2.91 | 81.3 |
| Standard Error |  | 0.12 | 1.1 | 13 | 0.17 | 58 | 0.16 | 0.9 |
| *Panicum bisulcatum* | C_3_ | 2.6 | 7.8 | 333 | 1.57 | 416 | 3.80 | 87.7 |
| *Panicum milioides* | C_3_-C_4_ | 2.2 | 7.4 | 297 | 1.24 | 387 | 3.22 | 92.3 |
| *Panicum amarum* | C_4_ | 3.2 | 33.1 | 97 | 0.86 | 800 | 1.08 | 89.5 |
| *Panicum antidotale* | C_4_ | 3.9 |  |  |  |  |  | 74.5 |
| *Panicum coloratum* | C_4_ | 3.4 | 11.1 | 306 | 1.59 | 445 | 3.61 | 84.8 |
| *Panicum deustum* | C_4_ | 5 | 15.4 | 325 | 1.17 | 306 | 3.83 | 84.8 |
| *Panicum dichotomiflorum* | C_4_ | 3.1 | 36.3 | 85 | 1.41 | 1540 | 0.92 | 92.6 |
| *Panicum milliaceum* | C_4_ | 2.1 | 7.2 | 292 | 1.13 | 313 | 3.65 | 79.9 |
| *Panicum monticola* | C_4_ | 5.3 | 18.2 | 291 | 1.97 | 543 | 3.67 | 79.4 |
| *Panicum phragmitoides* | C_4_ | 2.8 | 25.1 | 112 | 0.707 | 687 | 1.04 | 107 |
| *Panicum virgatum* | C_4_ | 3.3 | 12.7 | 260 | 0.854 | 271 | 3.15 | 82.6 |
| Mean |  | 3.35 | 17.4 | 240 | 1.25 | 571 | 2.80 | 86.6 |
| Standard Error |  | 0.31 | 3.38 | 32 | 0.12 | 120 | 0.40 | 2.9 |
| *Amaranthus edulis* | C_4_ | 4.14 | 18.2 | 227 | 0.847 | 289 | 2.94 | 77.5 |
| *Amaranthus hybridus* | C_4_ | 3.8 | 16 | 238 | 1.85 | 640 | 2.97 | 80 |
| *C. dactylon* | C_4_ |  | 21 |  |  | 402 |  | 89.2 |
| *Cenchrus ciliaris* | C_4_ | 6 | 19 | 316 | 2.1 | 470 | 4.52 | 69.9 |
| *Chrysanthellum indicum* | C_4_ | 4.7 | 28.1 | 167 | 1.21 | 598 | 2.03 | 82.4 |
| *Echinochloa crus-galli* | C_4_ |  | 18.4 |  |  |  |  | 83 |
| *Eragrostis tef* | C_4_ | 7.1 | 34.9 | 203 | 1.46 | 640 | 2.29 | 89 |
| *Megathyrsus maximus* | C_4_ | 5.3 | 13.9 | 381 | 1.25 | 265 | 4.75 | 80.3 |
| *P. dilatatum* | C_4_ |  | 19.9 |  |  | 415 |  | 88 |
| *Potulaca oleraca* | C_4_ | 5.9 | 13.6 | 434 |  |  | 5.56 | 78 |
| *Saccharum officinarum* | C_4_ | 3.9 | 26.3 | 148 |  |  | 1.80 | 82.2 |
| *Setaria italica* | C_4_ |  | 32.1 |  |  |  |  | 58 |
| *Setaria viridis* | C_4_ | 5.67 | 18.1 | 313 | 2.77 | 619 | 4.31 | 72.7 |
| *Sorghum bicolor* | C_4_ | 5.4 | 29.9 | 181 |  |  | 2.58 | 70 |
| *Urochloa mosambicensis* | C_4_ | 5.7 | 14.8 | 385 | 2.14 | 464 | 4.67 | 82.5 |
| *Urochloa panicoides* | C_4_ | 5.6 | 15.4 | 364 | 2.04 | 444 | 4.64 | 78.3 |
| *Z. japonica* | C_4_ |  | 18.5 |  |  | 403 |  | 84.1 |
| *Zea mays* | C_4_ | 4.19 | 30.6 | 137 | 0.920 | 59675 | 1.68 | 81.6 |
| Mean |  | 5.18 | 21.6 | 269 | 1.66 | 480000 | 3.44 | 79.0 |
| Standard Error |  | 0.27 | 1.6 | 28 | 0.20 | 36 | 0.37 | 1.8 |
|  |  |  |  |  |  |  |  |  |

**Appendix**

**Derivation of the kinetic equations**: The concentrations used in the kinetic equations are *E*: activated form of the enzyme; *R*: unbound RuBP; *ER*: RuBisCO…RuBP complex; *ER**: RuBisCO…enediolate of RuBP complex; *C*: free CO_2_; *O*: free O_2_; *ERC*: RuBisCO…carboxylated intermediate complex; *ERO*: RuBisCO…oxygenated intermediate complex; *ERP*: RuBisCO…carboxylated product complex; *ERX*: RuBisCO…oxygenated product complex; *G*: 3-phosphoglyceric acid; *Q*: 2-phospho-glycolate.

The mass balance equation for the kinetic mechanism (Fig. 1) is given by (*E_t_* is the total activated enzyme concentration)

*E* + *ER* + *ER** + *ERC* + *ERO* + *EP* + *EX* – *E_t_* = 0 (A1)

The steady state ordinary differential equations (ODEs) for this kinetic scheme are

$${d ER}/{dt=k_{1}}E\cdot R-\left( k_{2}+k_{3} \right)ER+k_{4}{ER}^{*}=0$$

(A2)

$${d {ER}^{*}}/{dt=k_{3}}ER-\left( k_{4}+k_{5}C+k_{11}O \right){ER}^{*}+k_{6}ERC+k_{12}ERO=0$$

(A3)

${d ERC}/{dt=k_{5}}{C\cdot ER}^{*}-\left( k_{6}+k_{7} \right)ERC+k_{8}EP=0$ (A4)

${d EP}/{dt=k_{7}}ERC-\left( k_{8}+k_{9} \right)EP+k_{10}E\cdot G\cdot G=0$ (A5)

${d ERO}/{dt=k_{11}}{O\cdot ER}^{*}-\left( k_{12}+k_{13} \right)ERO+k_{14}EX=0$ (A6)

${d EX}/{dt=k_{13}}ERO-\left( k_{14}+k_{15} \right)EX+k_{16}E\cdot G\cdot Q=0$ (A7)

It is convenient to define the following constants:

$\alpha_{C}=\left( k_{9}+k_{8} \right)/{k_{7}}$

$\alpha_{O}=\left( k_{15}+k_{14} \right)/{k_{13}}$

$\beta_{C}=k_{9}+k_{6}\alpha_{C}$

$\beta_{O}=k_{15}+k_{12}\alpha_{O}$

From the above steady state ODEs we can readily express the concentrations of free enzyme *E* and all reaction intermediates in terms of the product complexes (either *EP* or *EX*). For the carboxylation reaction (*EP*) we obtain (assuming only that product release is “irreversible” i.e.):

Summing (A2) to (A7): $E=\frac{k_{2}ER+k_{9}EP+k_{15}EX}{k_{1}R}$ (A8)

From (A5): *ERC* = *EP* (A9)

From (A7): *ERO* = *EX* (A10)

From (A4) + (A5) and (A9): $ER*=\frac{k_{9}EP+k_{6}ERC}{k_{5}C}=\frac{\beta_{C}}{k_{5}C}EP$ (A11)

From (A6) + (A7), (A10) and (A11): $EX=\frac{k_{11}O\cdot ER*}{\beta_{O}}=\frac{\beta_{C}k_{11}O}{\beta_{O}k_{5}C}EP$ (A12)

From (A10) and (A12):$ERO=\alpha_{O}EX=\frac{\alpha_{O}\beta_{C}k_{11}O}{\beta_{O}k_{5}C}EP$ (A13)

From (A2), (A8), (A11) and (A12):$ER=\frac{k_{9}EP+k_{15}EX+k_{4}ER*}{k_{3}}$

$=\frac{k_{9}}{k_{3}}EP+\frac{\beta_{C}k_{11}k_{15}O}{\beta_{O}k_{3}k_{5}C}EP+\frac{k_{4}\beta_{C}}{k_{3}k_{5}C}EP$ (A14)

Substituting (A8)-(A14) into (A1) and factorizing we get the steady state equation in the form

$\frac{E_{t}}{EP}=1+f_{1}^{C}+\frac{f_{2}^{C}}{R}+\frac{f_{3}^{C}}{C}+\frac{f_{4}^{C}}{RC}$ (A15)

where the coefficients$f_{i}^{C}$are given by

$f_{1}^{C}=\frac{k_{9}+k_{3}\alpha_{C}}{k_{3}}$ (A16a)

$f_{2}^{C}=\frac{k_{9}(k_{2}+k_{3})}{k_{1}k_{3}}$ (A16b)

$f_{3}^{C}=\frac{\beta_{C}}{k_{3}k_{5}}\left(k_{3}+k_{4}+\frac{(k_{3}+k_{15}+k_{3}\alpha_{O})k_{11}O}{\beta_{O}}\right)$ (A16c)

$f_{4}^{C}=\frac{\beta_{C}}{k_{1}k_{3}k_{5}}\left(k_{2}k_{4}+\frac{(k_{2}+k_{3})k_{11}k_{15}O}{\beta_{O}}\right)$ (A16d)

The rate of CO_2_ consumption is given by

$V_{C}=-\frac{dC}{dt}=k_{5}C\cdot ER*-k_{6}ERC=(k_{9}+k_{6}\alpha_{C})EP-k_{6}\alpha_{C}EP=k_{9}EP$ (A17)

Rewriting (A15) in terms of$EP$ and substituting the result into (A17) gives

$V_{C}=\frac{k_{9}E_{t}RC}{f_{4}^{C}+f_{3}^{C}R+f_{2}^{C}C+(f_{1}^{C}+1)RC}$ (A18)

When both substrates, *R* and *C*, are saturating the maximum rate of CO_2_ consumption,$V_{\max}^{C}$, is obtained as,

$V_{\max}^{C}=\frac{k_{9}}{1+f_{1}^{C}}E_{t}=k_{cat}^{C}E_{t}$ (A19)

Substituting (A16a) into (A19) and rearranging we get for $k_{cat}^{C}$ in terms of the rate constants$k_{i}$

$$k_{cat}^{C}=\frac{k_{3}k_{7}k_{9}}{k_{3}k_{7}+k_{3}k_{8}+k_{3}k_{9}+k_{7}k_{9}}$$

Finally, rewriting (A18) in terms of $V_{\max}^{C}$ gives the familiar (e.g. ([Farquhar, 1979](#_ENREF_10))) general form of the steady state rate equation,

$V_{C}=\frac{V_{\max}^{C}R\cdot C}{K_{3}^{C}+K_{2}^{C}R+K_{1}^{C}C+R\cdot C}$ (A20)

where $K_{i}^{C}=f_{i+1}^{C}/(1+f_{1}^{C})$. (A21)

It immediately follows that the rate of oxygen consumption by the enzyme can be written as

$$V_{O}=\frac{V_{\max}^{O}R\cdot O}{K_{3}^{O}+K_{2}^{O}R+K_{1}^{O}O+R\cdot O}$$

where $V_{\max}^{O}=k_{cat}^{O}E_{t}$ and $k_{cat}^{O}=\frac{k_{15}}{1+f_{1}^{O}}=\frac{k_{3}k_{13}k_{15}}{k_{3}k_{13}+k_{3}k_{14}+k_{3}k_{15}+k_{13}k_{15}}$.

The Michaelis-Menten equation (A20) for the single substrate *C* when *R* is saturating becomes

$$V_{C}=\frac{V_{\max}^{C}C}{K_{2}^{C}+C}$$

From (A21), (A16a) and (A16c):

$K_{2}^{C}=\frac{f_{3}^{C}}{1+f_{1}^{C}}=\frac{\beta_{C}}{(1+f_{1}^{C})k_{3}k_{5}}\left[k_{3}+k_{4}+\frac{(k_{3}+k_{15}+k_{3}\alpha_{O})k_{11}O}{\beta_{O}}\right]$ (A22)

Substituting$f_{1}^{O}=\frac{k_{15}+k_{3}\alpha_{O}}{k_{3}}$, $K_{R}=\frac{k_{3}}{k_{3}+k_{4}}$, $\gamma_{C}=\frac{\alpha_{C}}{1+f_{1}^{C}}$ and $\gamma_{O}=\frac{\alpha_{O}}{1+f_{1}^{O}}$

into (A22) yields the Michaelis constant in the presence of the other (*O*) substrate

$$K_{2}^{C}=K_{C}\left(1+\frac{O}{K_{O}}\right)$$

where $K_{C}=\frac{k_{9}+k_{6}\alpha_{C}}{(1+f_{1}^{C})K_{R}k_{5}}=\frac{k_{cat}^{C}+\gamma_{C}k_{6}}{K_{R}k_{5}}$ (A23)

and $K_{O}=\frac{k_{15}+k_{12}\alpha_{O}}{(1+f_{1}^{O})K_{R}k_{11}}=\frac{k_{cat}^{O}+\gamma_{O}k_{12}}{K_{R}k_{11}}$ (A24)

The specificities of each of the reactions are then

$S_{C}=\frac{k_{cat}^{C}}{K_{C}}=\frac{k_{cat}^{C}K_{R}k_{5}}{k_{cat}^{C}+\gamma_{C}k_{6}}$ and $S_{O}=\frac{k_{cat}^{O}}{K_{O}}=\frac{k_{cat}^{O}K_{R}k_{11}}{k_{cat}^{O}+\gamma_{O}k_{12}}$

and so the specificity of carboxylation relative to oxygenation (relative specificity) is given by

$S_{C/O}=\frac{S_{C}}{S_{O}}=\frac{k_{5}k_{cat}^{C}(k_{cat}^{O}+\gamma_{O}k_{12})}{k_{11}k_{cat}^{O}(k_{cat}^{C}+\gamma_{C}k_{6})}$ (A25)

In terms of the rate constants we find the coefficients of $k_{6}$ and $k_{12}$:

$\gamma_{C}=\frac{\alpha_{C}}{1+f_{1}^{C}}=\frac{(k_{8}+k_{9})k_{cat}^{C}}{k_{7}k_{9}}=\frac{k_{3}k_{8}+k_{3}k_{9}}{k_{3}k_{7}+k_{3}k_{8}+k_{3}k_{9}+k_{7}k_{9}}$ (A26)

$\gamma_{O}=\frac{\alpha_{O}}{1+f_{1}^{O}}=\frac{(k_{14}+k_{15})k_{cat}^{O}}{k_{13}k_{15}}=\frac{k_{3}k_{14}+k_{3}k_{15}}{k_{3}k_{13}+k_{3}k_{14}+k_{3}k_{15}+k_{13}k_{15}}$ (A27)

Thus the range of $\gamma$ is limited to [0,1].
